# Essential role of protein kinase R in the pathogenesis of pulmonary veno-occlusive disease

**DOI:** 10.1172/jci.insight.193495

**Published:** 2025-08-21

**Authors:** Amit Prabhakar, Rahul Kumar, Meetu Wadhwa, Abhilash Barpanda, Joseph Lyons, Asavari Gowda, Simren Gupta, Ananyaa Arvind, Prajakta Ghatpande, Arun P. Wiita, Brian B. Graham, Giorgio Lagna, Akiko Hata

**Affiliations:** 1 Cardiovascular Research Institute, UCSF, San Francisco, California, USA.; 2 Lung Biology Center, Pulmonary and Critical Care Medicine, Zuckerberg San Francisco General Hospital, California, USA.; 3 Department of Radiology,; 4 Department of Laboratory Medicine, and; 5 Helen Diller Family Comprehensive Cancer Center, UCSF, San Francisco, California, USA.; 6 Chan Zuckerberg Biohub San Francisco, San Francisco, California, USA.; 7 Department of Biochemistry and Biophysics, UCSF, San Francisco, San Francisco, California, USA.

**Keywords:** Cell biology, Vascular biology, Cell stress

## Abstract

Pulmonary veno-occlusive disease (PVOD) is a rare and severe subtype of pulmonary arterial hypertension, characterized by progressive remodeling of small pulmonary arteries and veins with no therapies. Using a mitomycin C–induced (MMC-induced) rat model, we previously demonstrated that protein kinase R–mediated (PKR-mediated) integrated stress response (ISR) drives endothelial dysfunction and vascular remodeling. To determine whether PKR is the primary mediator of ISR and the pathogenesis, we treated control (Ctrl) and PKR-knockout (KO) mice with the same dose of MMC. Consistent with rat data, Ctrl mice displayed ISR activation, vascular remodeling, and pulmonary hypertension after MMC treatment, while KO mice showed none of these phenotypes. Proteomic analysis revealed that MMC-mediated ISR activation attenuated protein synthesis in Ctrl but not in KO mice. These findings underscore the critical role of PKR-dependent ISR activation and subsequent perturbation of proteostasis as central mechanisms driving PVOD pathogenesis and identify PKR as a promising therapeutic target.

## Abstract 2

This study investigates the role of protein kinase R (PKR)-mediated integrated stress response (ISR) in pulmonary veno-occlusive disease (PVOD), a severe form of pulmonary hypertension. Using a mitomycin C (MMC)-induced rat model, the researchers found that PKR activation leads to endothelial dysfunction and vascular remodeling. In PKR knockout (KO) mice, MMC treatment did not trigger these effects, suggesting PKR is a key mediator in the disease’s pathogenesis. Proteomic analysis showed that MMC-induced ISR inhibits protein synthesis in control mice but not in KO mice. These results highlight the importance of PKR-dependent ISR activation in PVOD and suggest PKR as a potential therapeutic target.

## Graphical abstract

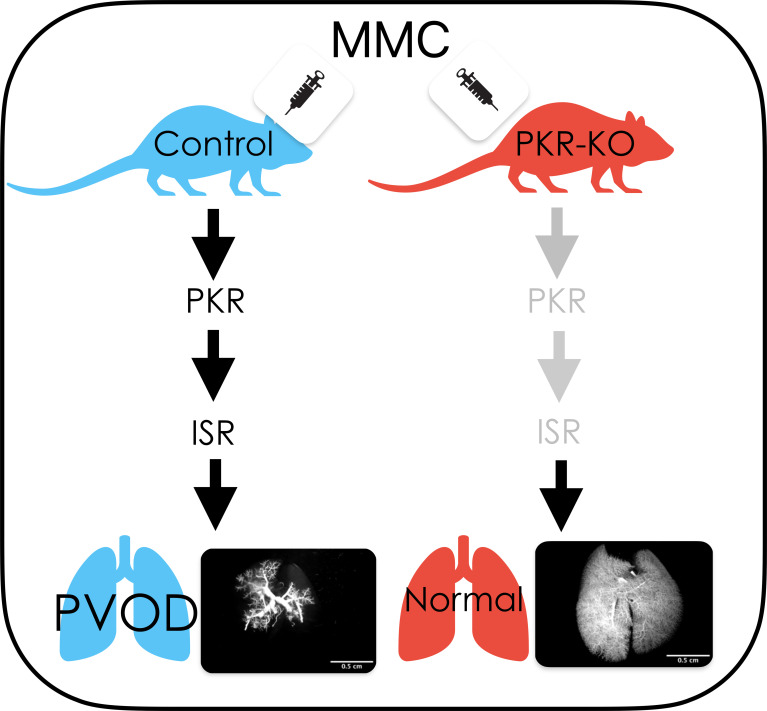

## Introduction

Pulmonary veno-occlusive disease (PVOD) is a severe form of pulmonary arterial hypertension (PAH), characterized by remodeling and obstruction of pulmonary arteries (PAs), pulmonary veins (PVs), and capillaries (1–3). The 5-year survival rate for patients with PVOD is 27% (4), lower than 61% for patients with PAH (5). PVOD affects both sexes equally and is estimated to occur in 1 in 5–10 million people per year (1–3). Due to the similarities in radiographic findings between PVOD and PAH, patients with PVOD are frequently misdiagnosed as having PAH (2, 3). Typically, patients with PVOD respond poorly to PAH therapies (2, 3). Furthermore, when treated with PAH-specific drugs, patients with PVOD can develop life-threatening pulmonary edema (2, 3). Thus, there is a critical need for therapeutics specifically tailored to PVOD.

Biallelic mutations in the *Eif2ak4* gene are the leading genetic cause of PVOD (6). *Eif2ak4* encodes general control nonderepressible 2 (GCN2, also known as EIF2AK4), 1 of the 4 eIF2 kinases, along with heme-regulated inhibitor (HRI, also known as EIF2AK1), protein kinase R (PKR, or EIF2AK2), and PKR-like endoplasmic reticulum kinase (PERK, also known as EIF2AK3), that become active in response to different cellular stresses and phosphorylate serine-51 (S51) of the α subunit of eukaryotic initiation factor 2 (eIF2α) (7). Upon phosphorylation of eIF2α, global translation is attenuated, thereby conserving energy, reprogramming gene expression, and restoring proteostasis, a physiological response to stress collectively known as the integrated stress response (ISR) (8, 9). The ISR is essential for an organism’s adaptation to stress conditions and changing environments (8, 9). When cap-dependent translation is globally suppressed, the transcripts with upstream open reading frames, such as cyclic AMP–dependent transcription factor 4 (ATF4) and growth arrest– and DNA damage–inducible protein 34 (GADD34; also known as PPP1R15A), are preferentially translated (8, 9). Upon activation of the ISR, the basic leucine zipper transcription factor ATF4 accumulates and translocates to the nucleus, where it binds to stress response genes (SRGs) and activates their transcription. The induction of SRGs facilitates organisms to manage stress, maintain fitness, and adapt to new environmental conditions (9).

In addition to the *Eif2ak4* mutations, exposure to alkylating agents, such as mitomycin C (MMC), cyclophosphamide, and cisplatin, is known to induce PVOD in cancer patients (10–12). Indeed, the administration of MMC to rats causes a spectrum of PVOD phenotypes, including right ventricular hypertrophy and pulmonary vascular lesions such as medial thickening, luminal obstruction in PAs and PVs, intimal obliteration, adventitial growth, and thrombosis. These features make it a valuable animal model for studying PVOD (11, 13–17). Using the MMC-induced PVOD model in rats, we demonstrated that MMC induces the activation of one of the eIF2 kinases, PKR, which subsequently triggers the ISR (16, 17). This leads to the depletion of the vascular endothelial adhesion molecule vascular endothelial cadherin (VE-Cad) in complex with Rad51, contributing to increased permeability and vascular remodeling (16). Administration of a small molecule inhibitor of PKR, C16, or an ISR inhibitor, ISRIB, prevents and attenuates ISR activation and PVOD pathogenesis in rats (16, 17). PKR regulates the innate immune response to viral infections (18). It is activated by binding to double-stranded RNA introduced during viral infection and replication (19). Several stimuli, including DNA damage, proinflammatory cytokines, and heat shock, are also known to activate PKR (19). The mechanism of PKR activation by MMC and the cell-type-specific responses downstream of the PKR/ISR axis remain unclear.

The potential involvement of the other 3 eIF2 kinases in activating the ISR and contributing to the development of cardiovascular phenotypes of PVOD in response to MMC remains unclear, given that all 4 eIF2 kinases are expressed in pulmonary vascular endothelial cells (PVECs) (16). In this study, we administered MMC to PKR homozygous null mice and examined the cardiopulmonary phenotypes associated with PVOD (20). Upon MMC treatment, WT littermates showed signs of active ISR and developed PVOD-like phenotypes as early as 5 days after treatment. In contrast, PKR-deficient mice did not exhibit signs of ISR activation or PVOD phenotypes after MMC treatment. This study reports the first murine model of PVOD to our knowledge and demonstrates that PKR is an essential mediator of ISR and the onset of PVOD-like vascular remodeling.

## Results

### No ISR activation following MMC treatment in PKR-deficient mice.

**Figure 1:**
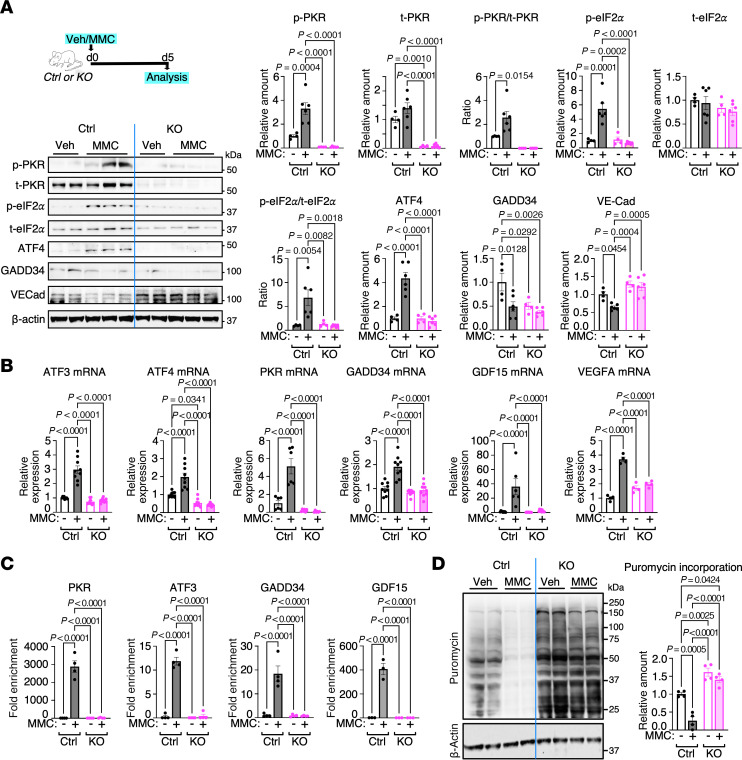
ISR activation upon MMC treatment is abolished in PKR-deficient mice. (**A**) Schematic representation of MMC-induced PVOD mouse model (top left) and immunoblot analysis of the indicated proteins in total lung lysates from vehicle (Veh)- or MMC-treated Control (Ctrl) and PKR-KO (KO) mice on day 5 (bottom left). The relative amounts of the indicated proteins, normalized to β-actin, are shown as mean ± SEM (right). *n* = 4 lung samples from Veh-treated Ctrl or KO mice and 6 lung samples from MMC-treated Ctrl or KO mice. (**B**) The levels of ATF4 target gene mRNAs, such as *ATF3*, *ATF4*, *PKR*, *GADD34*, *GDF15*, and *VEGFA*, in the lungs of Ctrl and KO mice treated with Veh or MMC on day 5 were analyzed by RT-qPCR. The results were normalized to β-actin levels and are presented as mean ± SEM. *n* = 9 independent samples. (**C**) ChIP assay was performed using the lungs harvested from Ctrl and KO mice treated with Veh or MMC employing an anti-ATF4 antibody, followed by PCR amplification corresponding to the genomic region of the *PKR*, *ATF3*, *GADD34*, and *GDF15* genes spanning the ATF4 binding sequence. The PCR results are presented as fold enrichment over the input as mean ± SEM. *n* = 4 independent experiments. (**D**) In vivo puromycin incorporation assay. Ctrl and KO mice treated with Veh or MMC were administered puromycin on day 5, followed by lung lysate preparation. After normalizing the total protein content, samples were subjected to SDS-PAGE. Puromycin-labeled proteins were visualized by immunoblotting using anti-puromycin and anti–β-actin antibodies (left panel). The levels of puromycin-labeled proteins, normalized to β-actin as a loading control, are shown as mean ± SEM (right panel). *n* = 4 independent samples per group. Statistical analysis was performed using 2-way ANOVA with Tukey’s multiple-comparison test.

To study the role of PKR in the MMC-mediated pathogenesis of PVOD, 9- to 10-week-old homozygous PKR-knockout (KO) mice in the C57BL/6 background, in which both copies of the *Eif2ak2* gene that encodes PKR were ablated (*Eif2ak2^–/–^*) (20, 21), and littermate control (Ctrl) mice (*Eif2ak2^+/+^*) were administered PBS (vehicle; Veh) or MMC. KO mice develop and breed with no phenotypes and have an average lifespan (20, 21). Five days after the administration of Veh or MMC, total lung lysates were subjected to immunoblotting to examine the activation status of PKR and ISR. The levels of active PKR were defined as the amount of PKR autophosphorylated at threonine-446 (p-PKR) relative to total PKR (t-PKR) (p-PKR/t-PKR ratio) and we found that it was increased 2.6-fold in MMC-treated Ctrl mice relative to Veh-treated Ctrl mice (Figure 1A), suggesting MMC induces PKR activation in Ctrl mice, like in rats (16, 17). Furthermore, the amount of the S51-phosphorylated eIF2α (p-eIF2α) relative to total eIF2α (t-eIF2α) (p-eIF2α/t-eIF2α ratio) and ATF4 were increased 1.3-fold and 4.3-fold in Ctrl mice following MMC treatment, respectively (Figure 1A). These results in Ctrl mice demonstrate that MMC mediates the activation of PKR and the ISR, which involves eIF2α phosphorylation and inhibition of cap-dependent translation, and preferential translation of ATF4 mRNA, similar to findings in rats (16, 17). As expected, p-PKR and t-PKR were below detectable levels in KO mice, validating the genetic ablation of PKR in KO mice (Figure 1A). When Ctrl mice were treated with the ISR inhibitor ISRIB [*trans*-*N*,*N*′-(cyclohexane-1,4-diyl)bis(2-[4-chlorophenoxy]acetamide)] (22, 23) or the PKR inhibitor C16 [6,8-dihydro-8-(1*H*-imidazol-5-ylmethylene)-7*H*-pyrrolo(2,3-g)benzothiazol-7-one] (24) in combination with MMC, the increases in the p-PKR/t-PKR ratio, the p-eIF2α/t-eIF2α ratio, and ATF4 expression were abolished, similar to the effect observed in rats (16, 17) (Supplemental Figure 1A; supplemental material available online with this article; https://doi.org/10.1172/jci.insight.193495DS1). MMC treatment reduced the amount of the regulatory subunit of protein phosphatase 1 (PP1), GADD34, which is essential for the dephosphorylation of eIF2α and inactivation of the ISR (Figure 1A) as observed in rats (17), indicating that MMC mediates constitutive eIF2α phosphorylation and ISR activation in Ctrl mice. The levels of mRNA (Supplemental Figure 1B) and protein (Supplemental Figure 1C) for other eIF2 kinases, such as GCN2, PERK, and HRI, were comparable between Ctrl and KO mice regardless of the treatments. In contrast with Ctrl mice, KO mice showed no change in the p-eIF2α/t-eIF2α ratio or ATF4 protein levels following MMC treatment, suggesting an absence of ISR activation in PKR-deficient mice (Figure 1A).

As previously observed in rats (16, 17), the levels of transcripts of the ATF4 target genes, such as *ATF3*, *ATF4*, *PKR*, *GADD34*, growth differentiation factor 15 (*GDF15*), and vascular endothelial growth factor A (*VEGFA*) (25–28), were increased 3.0-fold, 2.0-fold, 8.8-fold, 1.9-fold, 36.1-fold, and 3.7-fold, respectively, following MMC treatment in Ctrl mice (Figure 1B). Despite the increase in *GADD34* mRNA levels (Figure 1B), GADD34 protein levels decreased following MMC treatment in Ctrl mice (Figure 1A), similar to findings in rats (16, 17). This suggests that posttranscriptional regulation of *GADD34* leads to a reduction in GADD34 protein levels after MMC treatment. In contrast with Ctrl mice, no induction of ATF4 target gene transcripts was observed in KO mice following MMC treatment (Figure 1B). Mice heterozygous for PKR (*Eif2ak2^+/–^*) displayed a reduced induction of ATF4 target gene transcripts, suggesting that PKR haploinsufficiency leads to a partial induction of ATF4 target genes (Supplemental Figure 2). The chromatin immunoprecipitation with sequencing (ChIP-seq) experiment for ATF4 by the ENCODE consortium revealed the genome-wide ATF4 binding sites in human lymphoblast K562 cells (accession nos. ENCFF484GNY and ENCFF742FPU), which include the *PKR*, *ATF3*, *GADD34*, and *GDF15* genes (Supplemental Figure 3). After the administration of Veh or MMC, we performed a ChIP assay using the total lung lysates harvested from Ctrl mice and confirmed that the genomic fragments of *PKR*, *ATF3*, *GADD34*, and *GDF15* containing the ATF4 binding site (Supplemental Figure 3) were enriched 2,875-fold, 12-fold, 18-fold, and 355-fold, respectively, in MMC-treated Ctr mice over Veh-treated Ctrl mice (Figure 1C). However, we did not detect an association of ATF4 with any of these genes following MMC treatment in KO mice (Figure 1C), which is consistent with no induction of the *PKR*, *ATF3*, and *GADD34* transcripts in KO mice upon MMC treatment (Figure 1B). These results demonstrate that PKR is essential for the MMC-mediated induction of ATF4 and the transcriptional activation of ATF4 target genes. The in vivo puromycin incorporation assay revealed a 74% reduction in nascent protein synthesis in the lung of Ctrl mice following MMC treatment (Figure 1D), validating the ISR activation (8, 9). In KO mice, however, there was no reduction in the nascent protein synthesis after MMC treatment, confirming no ISR activation upon MMC treatment in KO mice (Figure 1D). We noted the levels of protein synthesis in Veh-treated KO mice were 1.6-fold higher than those in Veh-treated Ctrl mice, indicating that the deletion of PKR slightly increases the global protein synthesis (Figure 1D). These results demonstrate that PKR is responsible for the MMC-mediated ISR activation and attenuation of global protein synthesis.

### PKR-deficient mice do not develop PVOD.

**Figure 2:**
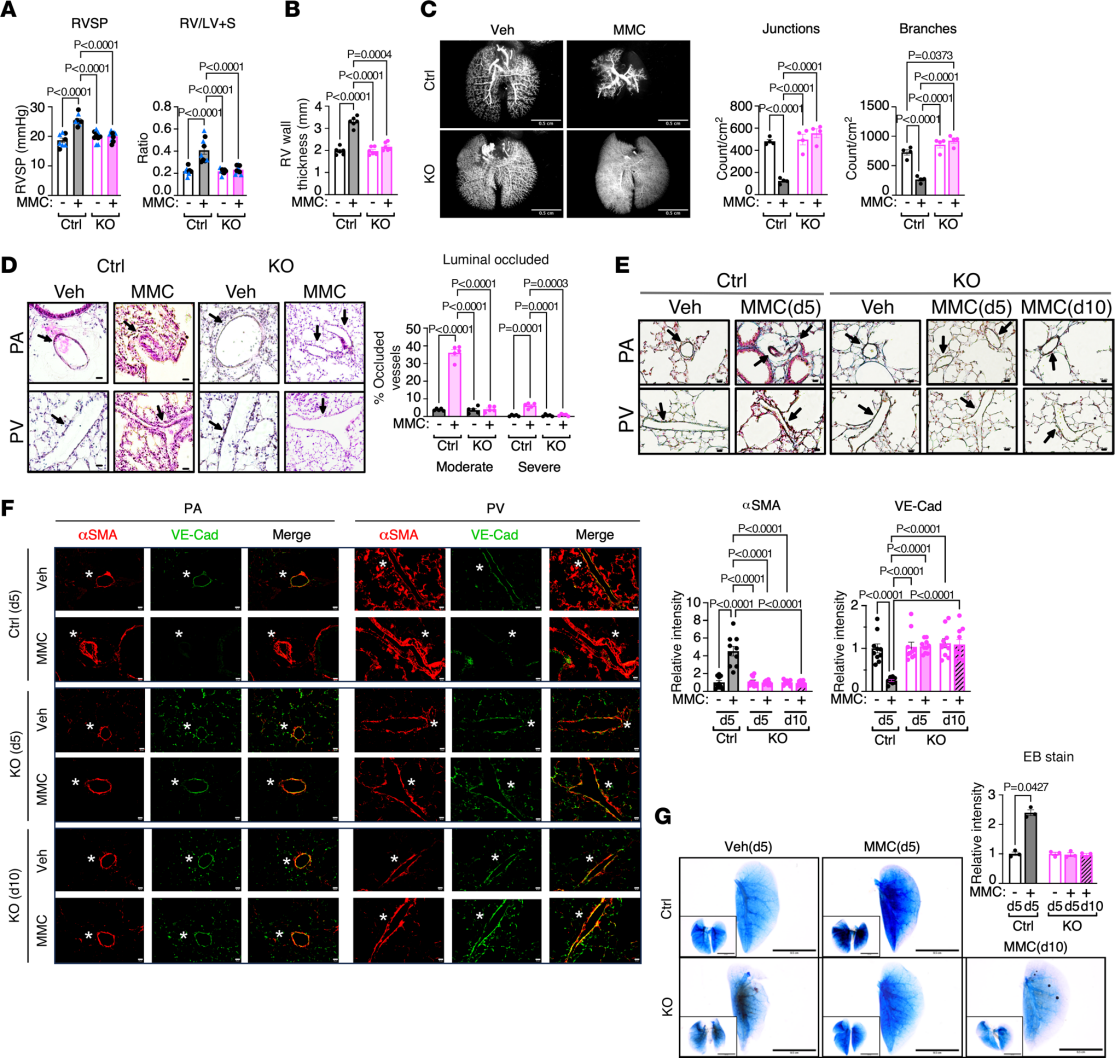
PKR-deficient mice do not develop PVOD phenotypes. (**A**) RVSP (mmHg) and RV/LV+S ratio in vehicle- (Veh) or MMC-administered control (Ctrl) and KO mice. Male and female animals are indicated as black circles and blue triangles, respectively. *n* = 8–9 independent samples per group. (**B**) RV wall thickness (mm) of Veh- or MMC-treated Ctrl and KO mice was measured and shown as mean ± SEM (left). The wall thickness was measured at 5 locations, and the mean value was calculated per sample. *n* = 6 independent samples per group. (**C**) Microfil casting of the lung vasculature in Ctrl and KO mice treated with either Veh or MMC on day 5 (d5). Holistic images of the entire lung are displayed (left). Scale bars: 0.5 cm. The number of junctions and branches per cm² of distal pulmonary vessels was quantified, with the data presented as mean ± SEM (right). *n* = 4 independent samples per group. (**D**) H&E staining of pulmonary vessels (PA and PV; arrows) in Ctrl and KO mice administered Veh or MMC (left). The third column is a magnified image of the black rectangle area in the second column (left). The fraction (%) of moderately (25%–40% occlusion) and severely (>40% occlusion) occluded vessels were counted and shown as mean ± SEM (right). Scale bars: 10 μm. *n* = 5 independent samples. (**E**) MSB staining visualizing collagens (blue) and smooth muscle cells (pink) in the lungs of Ctrl (on d5) and KO mice (on d5 and d10) following Veh or MMC administration. Scale bars: 10 μm. (**F**) PAs and PVs from Ctrl and KO mice on d5 or d10 after vehicle or MMC administration were stained with an anti-αSMA (red) and anti–VE-Cad (green) antibody for smooth muscle cells and endothelial cells, respectively. The merged images of αSMA and VE-Cad staining are shown. An asterisk indicates the location of the vessel (top). Scale bars: 50 μm. The signal intensities of αSMA and VE-Cad are quantified and are presented as mean ± SEM (bottom). (**G**) The permeability of pulmonary vasculature was assessed by injecting EB dye in Ctrl and KO mice administered with vehicle or MMC. The lung was harvested on d5 or d10, and the image was taken when the lung became translucent (left). Representative images of the whole lung and the largest lobe are presented. Scale bar: 0.5 cm. The relative intensities of EB staining were quantified and are presented as mean ± SEM (right). Statistical analysis was performed using 1-way ANOVA with Tukey’s multiple-comparison test (**A**) or 2-way ANOVA with Tukey’s multiple-comparison test (**B**–**D**, **F**, and **G**).

To examine whether MMC mediates pulmonary hypertension in the absence of PKR, Ctrl and KO mice were administered Veh or MMC. On day 5 after the MMC injection, we measured the right ventricular systolic pressure (RVSP) for PA pressure and the ratio of the RV weight to the left ventricle (LV) plus septum (S) weight (RV/LV+S) for RV hypertrophy. Ctrl mice treated with MMC exhibited an increase in RVSP, rising from 18.6 ± 0.7 mmHg to 25.4 ± 0.6 mmHg. In contrast, RVSP in KO mice remained unchanged following MMC treatment (19.8 ± 0.6 mmHg [Veh] vs. 19.9 ± 0.5 mmHg [MMC]) (Figure 2A). The basal and MMC-induced RVSP were comparable between male (black circles) and female (blue triangles) mice (Figure 2A), like the observations in rats (16, 17). Following MMC treatment, the RV/LV+S ratio increased in Ctrl mice from 0.22 ± 0.01 (Veh) to 0.41 ± 0.03 (MMC) (Figure 2A). In KO mice, however, the RV/LV+S ratio remained unchanged (0.22 ± 0.01 [Veh] to 0.23 ± 0.01 [MMC]) (Figure 2A), indicating no development of RV hypertrophy in KO mice after MMC. Deletion of PKR did not affect RVSP or the RV/LV+S ratio, as no differences were observed in these parameters between Veh-treated Ctrl and KO mice (Figure 2A). We also observed pleural and pericardial effusion, conditions associated with patients with PVOD (2), in all MMC-treated Ctrl mice. However, none of the MMC-treated KO mice developed pleural or pericardial effusion. Fifty percent of MMC-treated Ctrl mice died by day 8. In contrast, no mortality was observed in MMC-treated KO mice up to day 15 (Supplemental Figure 4), demonstrating the absence of MMC-induced cardiovascular phenotypes in KO mice. Ctrl mice, but not KO mice, showed a 67% increase in RV wall thickness following MMC treatment (Figure 2B and Supplemental Figure 5), confirming the development of RV hypertrophy in MMC-treated Ctrl mice. These results demonstrate that PKR-null mice are protected from developing the phenotypes of PVOD upon MMC administration. Microfil casting of vessels revealed obstructions in the distal pulmonary vessels of MMC-treated Ctrl mice (Figure 2C). Quantitative analysis demonstrated 74.4% and 63.5% fewer junctions and branches in the pulmonary vasculature of MMC-treated Ctrl mice compared with Veh-treated Ctrl mice, respectively (Figure 2C). In contrast, MMC treatment did not alter the number of junctions or branches in KO mice (Figure 2C). No remodeling of vasculature was observed in other organs, such as the brain, kidney, heart, liver, and intestine of Ctrl or KO mice following MMC treatment (Supplemental Figure 6), indicating that MMC-mediated vascular remodeling is specific to the lung like in MMC-rats (16, 17). Hematoxylin and eosin (H&E) staining of the lung showed that Ctrl mice developed medial hyperplasia and hypertrophy in PAs and PVs following MMC treatment (11, 13) (Figure 2D). The fraction of PAs and PVs with moderate (25%–40% occlusion) and severe (more than 40% occlusion) occlusion in Ctrl mice increased 9.5-fold (from 3.8% to 36.0%) and 14-fold (from 0.4% to 6.1%), respectively, following MMC treatment (Figure 2D). Ctrl mice that died on day 5 after the MMC administration revealed severe remodeling of PAs and PVs that resulted in complete occlusion of the lumen (Supplemental Figure 7A). KO mice did not develop vessel occlusion following MMC treatment (Figure 2D), which is consistent with the lack of increase in the RVSP and RV/LV+S ratio in MMC-treated KO mice (Figure 2A). There was no spontaneous development of vascular remodeling in Veh-treated KO mice (Figure 2D). Martius scarlet blue (MSB) staining of the lungs revealed the increased thickness of the smooth muscle layer (pink stain) and the accumulation of collagens (blue stain) in the PAs and PVs of MMC-treated Ctrl mice on day 5 (Figure 2E). However, no changes were observed in PAs and PVs of MMC-treated KO mice on day 5 or day 10 after MMC treatment when greater than 70% of Ctrl mice had died (Figure 2E). MSB staining of lung tissues from Ctrl mice that died on day 5 following MMC administration revealed abnormal thickening of the smooth muscle layer and collagen accumulation in both PAs and PVs (Supplemental Figure 7B). Erythrocyte and platelet accumulation was observed within the lumens of the PAs (Supplemental Figure 7B). Immunofluorescent (IF) staining of PAs and PVs was performed using antibodies against VE-cadherin (VE-Cad), a marker of vascular endothelial cells, and α-smooth muscle actin (αSMA), a marker of smooth muscle cells. IF analysis revealed a 4.5-fold increase in αSMA signal by day 5 following MMC treatment in Ctrl mice, indicating medial layer thickening; this change was not observed in KO mice (Figure 2F). In MMC-treated Ctrl mice, VE-Cad signal was reduced to 25% of that in Veh-treated Ctrl mice, suggesting the depletion of endothelial cells. In contrast, VE-Cad signal remained unchanged in KO mice after MMC (Figure 2F). Notably, 10 days after MMC treatment in KO mice, αSMA and VE-Cad signals remained comparable to baseline, confirming no evidence of medial thickening or obliteration of endothelium following MMC treatment in KO mice (Figure 2F). Immunoblot analysis of p-PKR, p-eIF2α, and ATF4 showed no evidence of ISR activation in MMC-treated KO mice on day 10, in contrast with control mice (Supplemental Figure 8A). There was no increase in *ATF3* or *GDF15* mRNA levels, both transcriptional targets of ATF4, in MMC-treated KO mice on day 10 (Supplemental Figure 8B). These findings are consistent with the absence of vascular remodeling observed in MMC-treated KO mice on day 10 (Figure 2, E and F) and support the notion that the activated ISR pathway drives vascular remodeling. When the vasculature of Ctrl mice was perfused with Evans blue (EB) dye, EB staining intensity was increased 2.4-fold after MMC treatment (Figure 2G), indicating that MMC treatment compromised the endothelial barrier, increased vascular permeability, and led to EB dye extravasation. In contrast, no increase in EB staining was detected in KO mice on day 5 or day 10 after MMC (Figure 2G), confirming that PKR plays an essential role in MMC-induced impairment of the pulmonary vascular barrier and increased permeability. Immunostaining for CD68 indicated an elevated number of monocytes and macrophages infiltrated into the pulmonary vessels of MMC-treated Ctrl mice, but not in MMC-treated KO mice (Supplemental Figure 9). These findings demonstrate that, similar to rats (16, 17), MMC administration in mice induces PVOD phenotypes that overlap with those observed in patients with PVOD. Furthermore, the absence of PKR prevents MMC-induced ISR activation, pulmonary vascular remodeling, induction of fibrosis, and monocyte/macrophage infiltration, confirming a causal role of the MMC/PKR/ISR axis in the pathogenesis of PVOD. The findings in KO mice rule out the possibility that the therapeutic effects of C16 in the MMC-induced PVOD (16, 17) are due to off-target actions. Additionally, these findings exclude the involvement of other translation regulatory mechanisms, such as mammalian target of rapamycin complex 1 (mTORC1) (29).

### PKR-depleted vascular endothelial cells are not impaired by MMC treatment.

**Figure 3:**
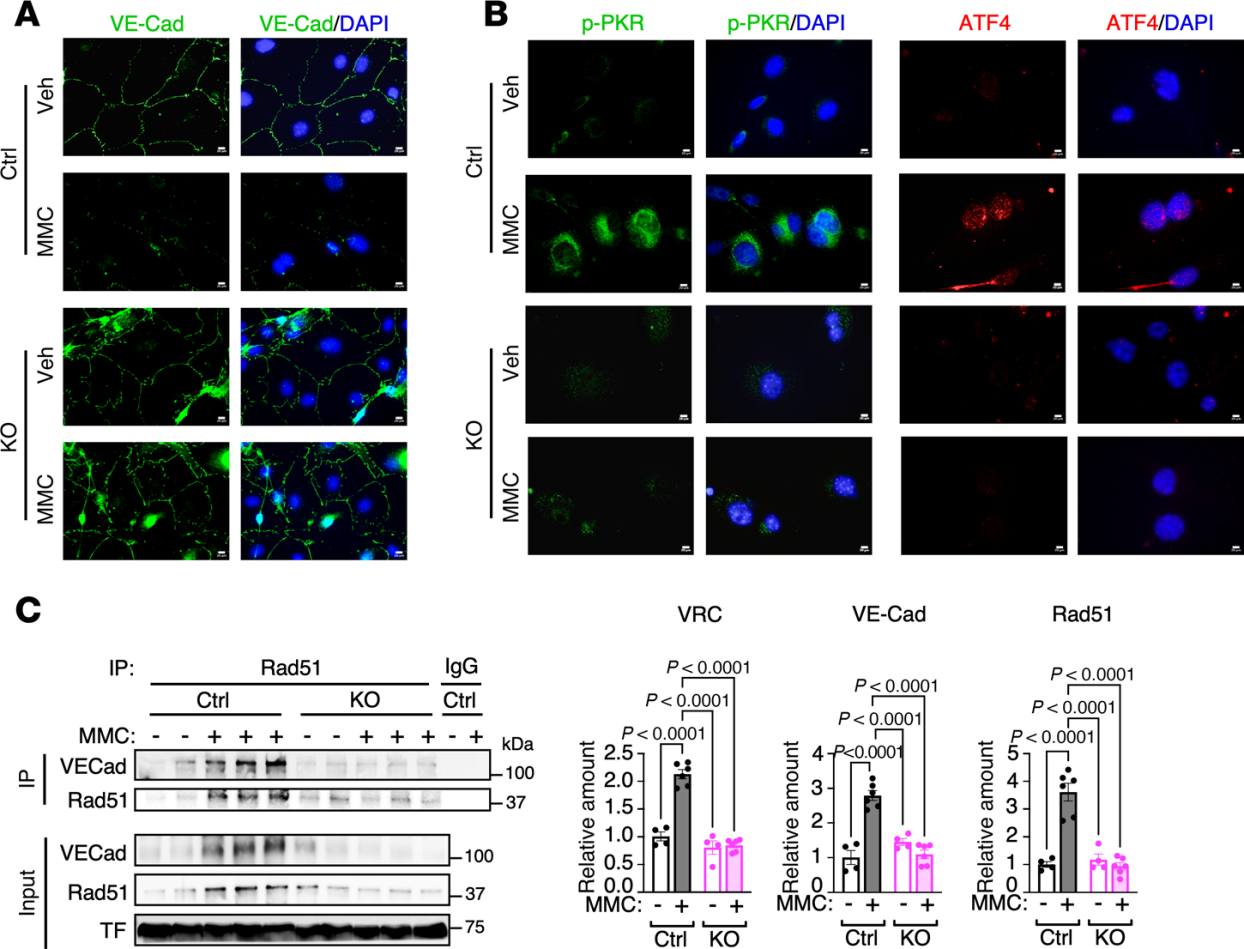
MMC treatment does not impair pulmonary vascular endothelium in PKR-deficient mice following MMC treatment. (**A**) PVECs were isolated from the lungs of Ctrl and KO mice administered with vehicle or MMC and subjected to IF staining with anti–VE-Cad antibody (green) and DAPI (blue) for nuclei. Scale bars: 10 μm. (**B**) PVECs isolated from the mice administered vehicle or MMC were subjected to IF staining for p-PKR (green, left), ATF4 (red, right), and with DAPI (blue) for nuclei. Scale bars: 10 μm. (**C**) The plasma isolated from Veh- or MMC-treated Ctrl and KO mice on day 5 were subjected to IP with an anti-Rad51 antibody or nonspecific IgG (control), followed by immunoblot analysis with an anti–VE-Cad (for VRC) and anti-Rad51 antibody to detect the interaction between these proteins. Immunoblot with an anti-transferrin antibody (TF) is shown as loading control. *n* = 2 plasma samples from Veh-treated Ctrl or KO mice and 3 plasma samples from MMC-treated Ctrl or KO mice (left). The relative amounts of the indicated proteins normalized to TF are demonstrated as mean ± SEM (right). *n* = 4 plasma samples from Veh-treated Ctrl or KO mice and 6 plasma samples from MMC-treated Ctrl or KO mice (left). Statistical analysis was performed using 2-way ANOVA with Tukey’s multiple-comparison test.

We previously demonstrated that MMC treatment induces the depletion of the adherens junction protein VE-Cad (also known as Cdh5), resulting in increased vascular permeability (16, 17). To investigate this mechanism further, we isolated PVECs from the lungs of Ctrl and KO mice treated with either Veh or MMC. These cells were subjected to IF staining for VE-Cad. PVECs from MMC-treated Ctrl mice exhibited a reduction in VE-Cad levels. In contrast, PVECs from MMC-treated KO mice retained VE-Cad expression at levels comparable to those observed in Veh-treated Ctrl or KO mice (Figure 3A). Additionally, IF staining for p-PKR and ATF4 confirmed the activation of the ISR in PVECs from MMC-treated Ctrl mice, as evidenced by the induction of p-PKR in the cytoplasm and ATF4 in the nucleus (Figure 3B). In contrast, PVECs from MMC-treated KO mice showed neither p-PKR staining nor ATF4 accumulation, indicating the lack of ISR activation (Figure 3B). These results align with the immunoblotting results obtained from lung lysates (Figure 1A) and support the idea that the activation of PKR/ATF4/ISR axis following MMC treatment occurs in vascular endothelium. Moreover, these results demonstrate that MMC does not mediate the downregulation of VE-Cad and impairs the endothelial barrier when PKR is ablated; therefore, ISR activation is prevented in the vascular endothelium.

We previously demonstrated that the VE-Cad and Rad51 complex (VRC), located at the adherence junctions in PVECs, is depleted due to the release of VRCs into circulation following MMC treatment in rats (16, 17). By IP with an anti-Rad51 antibody, followed by immunoblotting with an anti–VE-Cad antibody, we found a 2.1-fold increase in the amount of VRC in the plasma of Ctrl mice upon MMC treatment (Figure 3C). Similarly to VRCs, the levels of VE-Cad and Rad51 in the input plasma samples of Ctrl mice increased by 2.8-fold and 3.6-fold, respectively, following MMC treatment (Figure 3C). In contrast, KO mice showed no induction of plasma VRC, VE-Cad, or Rad51 after MMC treatment (Figure 3C). These results demonstrate that the junctional structure of the pulmonary vascular endothelium in KO mice is protected from MMC-mediated damage because of the ablation of PKR-mediated ISR activation and the release of VRCs.

### Proteomic analysis reveals protection in KO mice from MMC-mediated translational inhibition.

**Figure 4:**
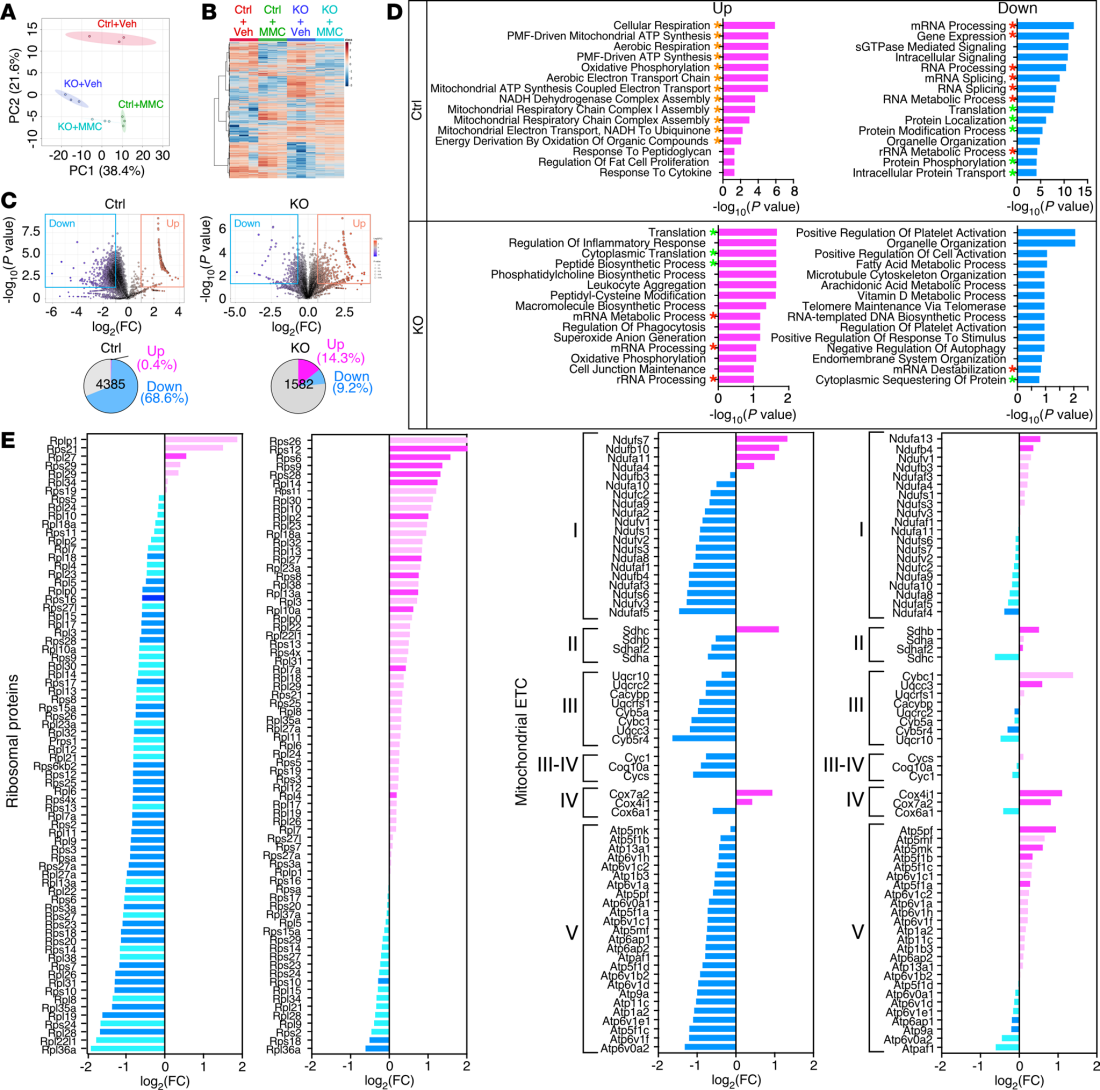
Effects of MMC on the lung proteomic landscape in Ctrl and KO mice. (**A**) Lungs harvested on day 5 from Veh- or MMC-treated Ctrl and KO mice were subjected to MS analysis. The principal component analysis of the MS data for lungs from Veh- or MMC-treated Ctrl and KO mice, conducted in triplicate, is shown. (**B**) A hierarchically clustered heatmap of differentially expressed proteins (DEPs) that are statistically significant (*P* < 0.05 by 2-tailed Student’s *t* test) in the lungs of Veh- or MMC-treated Ctrl and KO mice. *n* = 3 per group. (**C**) Volcano plots compare the proteome of MMC-treated versus Veh-treated Ctrl and KO mouse lungs (top). A larger circle size represents a lower *P* value, while the color gradient from blue to red corresponds to increasing log_2_(FC) values, with blue indicating lower values and magenta indicating higher values. Pie charts demonstrate the fraction (%) of DEPs upregulated (magenta) or downregulated (blue), with a threshold of log_2_(FC) greater than 0.8 or less than –0.8 in Ctrl and KO mice (bottom). The gray part represents the fraction of DEPs with –0.8 ≤ log_2_(FC) ≤ 0.8. *n* = 3 per group. (**D**) Top 15 pathways most enriched in upregulated (magenta) and downregulated (blue) DEPs in the lungs of Ctrl (top) and KO mice (bottom) following MMC treatment. The green, red, and orange asterisks indicate pathways related to protein synthesis/modifications, RNA metabolism, and mitochondrial ATP synthesis, respectively. (**E**) The bar graphs indicate the fold change in ribosomal proteins (left panel) and components of the mitochondrial electron transport chain (ETC) (right panel) between vehicle and MMC treatment in Ctrl and KO mice. Magenta and blue colors indicate upregulated and downregulated proteins, respectively. Darker colored bars represent *P* < 0.05. Differential expression of proteins was assessed using Welch’s *t* test.

One of the consequences of ISR activation is the attenuation of global cap-dependent translation due to eIF2α phosphorylation (8, 9). To examine the effect of PKR-mediated ISR activation on the protein dynamics upon MMC treatment, we applied quantitative mass spectrometry (MS) analysis and compared the lung proteome between Veh- and MMC-treated Ctrl and KO mice. Principal component analysis (PCA) (Figure 4A) revealed that the proteome of Veh- (Ctrl-Veh) and MMC-treated Ctrl (Ctrl-MMC) mice were highly distinct (Figure 4A). In contrast, the proteome of Veh-treated (KO-Veh) and MMC-treated KO mice (KO-MMC) were more closely associated (Figure 4A). Among the 7,598 proteins detected in the lungs of Ctrl and KO mice, 4,385 proteins in Ctrl mice and 1,583 proteins in KO mice were identified as differentially expressed, based on a *P*-value cutoff of 0.05 and a log_2_(FC) threshold of greater than 0.8 or less than –0.8. A hierarchically clustered heatmap of differentially expressed proteins (DEPs) illustrated changes in protein levels following MMC treatment and genetic ablation of PKR (Figure 4B). In Ctrl mice, 68.6% of DEPs (3,006 proteins) exhibited decreased abundance after MMC treatment, whereas only 9.2% of DEPs (145 proteins) showed a decrease in KO mice (Figure 4C). These observations support our results that, in KO mice, ISR activation and global translational attenuation were not triggered by MMC (Figure 1). In contrast, 0.4% (17 proteins) and 14.3% (226 proteins) of DEPs showed increases in Ctrl and KO mice, respectively, following MMC treatment (Figure 4C). Gene ontology (GO) enrichment analysis revealed 5 most overrepresented GO terms in DEPs downregulated in Ctrl mice were protein metabolism, including protein synthesis, modification, and localization (Figure 4D, green asterisks). We also noted that proteins associated with RNA metabolism were overrepresented in downregulated DEPs in Ctrl mice (Figure 4D, red asterisks). In KO mice, however, the GO terms overrepresented in downregulated DEPs in KO mice were divergent from those in Ctrl mice, such as platelet activation and the metabolic process of fatty acid, arachidonic acid, and vitamin D (Figure 4D). Indeed, 67 out of 73 ribosomal proteins (92%) were downregulated in Ctrl mice following MMC treatment, resulting from the attenuation of protein synthesis through ISR activation by PKR (Figure 4E). In contrast, only 21 out of 73 ribosomal proteins (29%) were downregulated in KO mice, presumably due to a lack of PKR/ISR activation (Figure 4E). We noted that the GO terms related to the mitochondrial electron transport chain (ETC) were overrepresented in DEPs upregulated in MMC-treated Ctrl mice (Figure 4D, orange asterisks). However, we found that 89% (56 out of 63) of the primary components of ETC complexes I–V decreased following MMC treatment in Ctrl mice (Figure 4E), which could result in reduced ATP synthesis and an accumulation of reactive oxygen species (30). In contrast, in KO mice, 57% (36 out of 63) of these proteins were upregulated following MMC treatment (Figure 4E). These results suggest that the MMC-mediated activation of the PKR/ISR axis results in robust proteomics changes, disrupts cellular homeostasis, and mediates vascular remodeling in PVOD. Moreover, genetic ablation of PKR protects against MMC-induced ISR activation, attenuation of protein synthesis, and PVOD pathogenesis. Thus, we identify PKR as a promising therapeutic target for PVOD.

## Discussion

In this study, we established a murine model of PVOD by administering MMC at the same dosage previously used to generate PVOD in Sprague-Dawley rats (16, 17). Like in rats, MMC-treated mice developed obliterative fibrosis in arterioles and venules, adventitial growth, and capillary hemangiomatosis, characteristics of PVOD patients (16, 17). Unlike in rats, where vascular remodeling becomes evident by day 8 and progresses to more severe manifestations by day 24, and the phenotypes remain the same up to day 60 with no mortality following MMC treatment (16, 17), Ctrl mice exhibited severe PVOD phenotypes, including pleural and pericardial effusion, by day 5. By day 14, all MMC-treated Ctrl mice died of severe pulmonary vascular remodeling in PAs and PVs. In contrast, no mortality was observed among MMC-treated KO mice, indicating the resilience of KO mice against the insult by MMC.

The biallelic mutation in the *Eif2ak4* gene, which encodes GCN2, is the primary genetic cause of PVOD (6). Our findings of the involvement of PKR activation in PVOD pathogenesis are seemingly at odds with the genetic observations that PVOD-associated mutations in *Eif2ak4* are believed to result in loss-of-function/expression mutations in GCN2, given that GCN2 and PKR share the same function (6, 16, 17). Recent studies on missense GCN2 mutants associated with PVOD show that some mutants are misfolded and degraded or kinase-inactive, while others are hypomorphic (31). Thus, it remains unclear whether all PVOD patients carrying *Eif2ak4* mutations develop the disease due to the inactivation of GCN2. It is plausible that genetic inactivation of GCN2 could lead to compensatory activation of PKR (8). The mechanisms driving the compensatory activation of PKR should be explored.

Here, we demonstrate that both pharmacological intervention and genetic ablation of PKR prevent MMC-induced ISR activation and the development of PVOD, thereby confirming the essential role of the PKR/ISR/ATF4 axis in PVOD pathogenesis. PKR was initially identified as a viral infection sensor that inhibits viral replication by suppressing host cell translation (19). In recent studies, however, PKR has been recognized to play a critical role in maintaining tissue homeostasis under sterile conditions (19). Mutations in PKR or its activator protein PACT, which result in increased PKR activation, are linked to the neurological disorder dystonia (32–34). Aberrant PKR activation has also been linked to autoimmune diseases, such as rheumatoid arthritis and systemic lupus erythematosus (19). Given the ubiquitous expression of PKR, examining the phenotypes of vascular endothelial cell–specific PKR-KO mice might provide further insight into the role of the PKR/ISR pathway, specifically in the endothelium.

We also provided evidence of the role of the PKR/ISR axis in the reprogramming of the proteome in vascular endothelial cells. It is well accepted that PKR, along with the other 3 eIF2 kinases, phosphorylates eIF2α and attenuates global cap-dependent translation as a critical component of the ISR (8, 9). The transient activation of the ISR and changes in proteostasis under stress are thought to enhance cellular resilience and facilitate adaptation to stress conditions (8, 9). However, the quantitative and qualitative changes in the proteome upon specific stress within a specific tissue have not been well characterized. Our proteomic analysis in Ctrl mice demonstrates that MMC treatment leads about a quarter of lung proteins to be up or downregulated more than 1.74-fold. Among the DEPs downregulated in Ctrl mice, proteins associated with protein and RNA metabolism were enriched following MMC treatment. MMC promotes constitutive eIF2α phosphorylation and translational inhibition in the pulmonary vascular endothelium of PVOD rats and patients through downregulating the PP1 complex that dephosphorylates Ser51 of eIF2α, which is where PKR phosphorylates upon MMC treatment (16, 17). We speculate that constitutive eIF2α phosphorylation and ISR activation, as opposed to transient ISR activation, lead to the irreversible reprogramming of proteostasis and drive the deregulation of endothelial cells and pathologic remodeling of pulmonary vessels in PVOD. Future studies focusing on the pathways enriched in DEPs in Ctrl mice (Veh vs. MMC) will reveal previously unappreciated mechanisms underlying vascular remodeling in PVOD.

A total of 68.6% of the proteins reduced in Ctrl mice following MMC treatment, as identified by proteomic analysis, is close to the observations in the puromycin incorporation assay, demonstrating a 74% reduction in nascent proteins. It is plausible that, under our experimental conditions, proteins with long half-lives are not notably reduced following MMC treatment. This could lead to an underestimation of the impact of global translational attenuation induced by the PKR/ISR axis in our proteomics analysis. Stable isotope labeling by amino acids in cell culture–based (SILAC-based) proteomic analysis and ribosome profiling might better represent proteome dynamics following ISR activation (35, 36). It is well established that cellular energy production and consumption are regulated by various environmental and physiological stressors (37, 38). Our results indicate that, following MMC treatment, most mitochondrial ETC components are downregulated in Ctrl mice. These findings suggest that PKR-dependent ISR activation reduces energy production by depleting ETC components while reducing energy consumption by suppressing RNA and protein metabolism. In conclusion, this study highlights the essential role of PKR in ISR activation and its impact on proteostasis, ultimately contributing to PVOD pathogenesis. Our findings suggest PKR inhibitors are promising candidates for therapeutic intervention in PVOD.

## Methods

Reagents, kits, antibodies, PCR primers, siRNAs, instruments, and software used in the study are listed in Supplemental Tables 1–4.

### Sex as a biological variable.

Sex was not considered a biological variable in this study. Both male and female animals were used in all experiments.

### Mouse model of PVOD.

*EIF2AK2*-knockout (KO) mice (21) were provided by Gokhan Hotamisligil (Harvard T.H. Chan School of Public Health, Boston, Massachusetts, USA). Ctrl and KO mice in the C57BL/6 background were housed in the vivarium of the cardiovascular research building at UCSF. Both male and female young mice (9–10 weeks old) were subjected to the following protocols to examine the effect of MMC. Two mg of MMC was dissolved in 1 mL phosphate-buffered saline (PBS). Mice were randomly divided into MMC-treated (3 mg/kg body weight) or vehicle-treated (PBS) groups. MMC or vehicle was administered once by intraperitoneal injection (i.p.) on day 0. Mice were euthanized on day 5 for hemodynamic measurements, RV hypertrophy assessment, and tissue collections.

### Simultaneous MMC and ISRIB or C16 treatment.

The ISR inhibitor ISRIB (Sigma-Aldrich, 19785; 0.25 mg/kg) or vehicle (DMSO) was administered to mice i.p. 30 minutes prior to MMC treatment (3 mg/kg) on day 0. ISRIB or vehicle was subsequently administered i.p. 3 times per week until day 5. The PKR inhibitor C16 (Sigma-Aldrich, SML0843; 100 μg/mL) was administered once at a dose of 33.5 μg/kg via i.p. injection. On day 5, lung tissue samples were also collected for further analysis.

### Hemodynamic assessment.

To assess hemodynamics, terminal right heart catheterization was performed in mice using an open-chest method, as previously described (39, 40). Mice were anesthetized i.p. with a mixture of ketamine (100 mg/kg) and xylazine (40 mg/kg). A tracheostomy tube was placed, and mechanical ventilation was initiated at a tidal volume of 6 cc/kg. Following anesthesia, the abdomen and diaphragm were carefully dissected to minimize blood loss. A 1-F pressure-volume catheter (PVR-1035, Millar AD Instruments) was inserted directly into the RV and subsequently into the LV through their respective free walls. At the end of the hemodynamic measurements, the lungs were perfused with PBS via the RV. The Fulton index, calculated as the RV/LV+S ratio, was used to measure RV hypertrophy. The left lung lobes were inflated with 1% agarose and fixed in 10% formalin for histological analysis, while the 4 right lung lobes were snap-frozen at –80°C for subsequent protein and RNA analysis.

### H&E staining.

Anesthetized mice were flushed with 1× PBS, fixed in 4% paraformaldehyde (PFA) (w/v), transferred to 1× PBS after 24 hours, and embedded in paraffin. The right bronchus of flushed lungs was sutured, the left lung inflated with 1% low-melt agarose for fixation and paraffin embedding, and the right lung split for snap freezing for protein and RNA studies.

To assess PA and PV muscularization, mouse lung tissue sections (10 μm in thickness) were stained with H&E. The external and internal diameter of a minimum of 50 transversally cut vessels in tissue blocks ranging from 25 to 80 μm was measured by determining the distance between the lamina elastica externa and lumen in 2 perpendicular directions, as described previously (41). The vessels were subdivided based on their diameter (microvessels, <50 μm and medium-sized vessels, 50–80 μm), and the assessment of muscularization was performed using ImageJ (NIH) in a blinded fashion by a single researcher to reduce operator variability, which was not aware of the group allocation of the samples being analyzed. The vessels with less than 40% luminal occlusion were considered moderately occluded, and those with greater than 40% were identified as severely occluded. The percentages represent the number of moderately or severely occluded vessels relative to the total number of vessels counted. Images were acquired using a Nikon Eclipse Ts2 inverted LED phase contrast microscope and a Leica SPE confocal microscope. Airways (bronchi and bronchioles) follow a branching pattern that mirrors the tree-like structure of the lung’s lobes and segments, while veins have a more variable course as they drain blood back to the heart. Airways generally have thicker walls compared with veins. When cut in cross section, they are more likely to maintain a round or oval shape, while veins may appear more collapsed or irregular. These characteristics were applied to distinguish veins from airways.

### MSB staining.

MSB staining was performed based on a previously described protocol (42). Briefly, paraffin-embedded mouse lung sections were deparaffinized and sequentially stained with iron hematoxylin for 5 minutes, Martius yellow for 3 minutes, and crystal scarlet solution for 3 minutes. Slides were rinsed with distilled water in between every step. Next, slides were dipped into phosphotungstic acid differentiator for 10 minutes followed by staining with aniline blue for 3 minutes. Tissues were dehydrated with absolute alcohol, cleared in xylene, and mounted. Images were acquired using a Nikon Ts2 microscope.

### CD68 immunohistochemistry.

Lung sections were deparaffinized, hydrated, and incubated in 10 mM sodium citrate buffer in a microwave oven for antigen retrieval. Sections were incubated overnight at 4°C with a primary antibody against CD68 (ab125212, Abcam). After extensive washing in PBS, sections were incubated with HRP-coupled secondary antibodies for 90 minutes and were visualized using 3,3-diaminobenzidine, counterstained with methyl green (H-3402, Vector Laboratories), and images were acquired using a Nikon Ts2 microscope.

### Perfusion of pulmonary vasculature with EB dye and the analysis of vascular permeability in vivo.

Perfusion of the pulmonary vasculature was performed as described previously (16, 43). Briefly, a 2% (weight/volume) EB solution was prepared by mixing EB (E2129, Sigma-Aldrich) in 0.9% NaCl, followed by filtering through a 0.22-μm filter. After the incision was made in the midline of the animal and the diaphragm was exposed, using forceps, the sternum was grabbed and pulled towards the head of the animal, pressing the heart against the diaphragm until it was easily visible. A 25-gauge needle with a 3 mL syringe was inserted through the diaphragm into the LV to deliver 2% EB solution (6 mL/g). After waiting for approximately 5 minutes to allow the EB to circulate, we confirmed the successful administration of the EB and proceeded to tissue harvest. The harvested tissue was fixed in 4% PFA overnight at 4°C. After washing in 1× PBS with shaking at room temperature 3 times for 30 minutes each, the tissue was dehydrated with a methanol/H_2_O series: 20%, 40%, 60%, 80%, 100%; 1 hour each. After further washing with 100% methanol for 1 hour, the tissue was incubated for 3 hours with shaking in 66% dichloromethane (270997, Sigma-Aldrich)/33% methanol at room temperature, followed by incubation in 100% dichloromethane 2 times, 15 minutes each, with shaking. The tissue was then incubated in 100% dibenzyl ether (108014, Sigma-Aldrich) until it became translucent and photographed. The permeability of the pulmonary vasculature was examined by quantifying the intensity of EB stain by ImageJ.

### Microfil casting of vasculature.

The procedure for casting the vessels with Microfil was described previously (44). Briefly, heparin (1000 IU/kg), as an anticoagulant, was injected intravenously 10 minutes before anesthesia. The mice were anesthetized using a ketamine/xylazine cocktail. After removing the anterior chest wall, a microperfusion tube was inserted and kept in the right ventricle via a needle (25 gauge) for perfusion with PBS, which drained from the left atrium. The lungs were perfused to clear all blood, as evidenced by turning the lung tissue white. During the perfusion of pulmonary PAs, a freshly dissolved Microfil polymer mixture (MV compound/MV diluent/MV agent = 5:5:1) was instilled via a 25-gauge needle inserted into the PA from the incision through the RV wall by manual injection. The Microfil mixture was gently infused into the PA under a dissecting microscope until it reached the terminal branches of PAs and stopped within 2–3 seconds. The lungs were then kept at room temperature for approximately 90 minutes or overnight at 4°C while covered with a wet paper towel to avoid desiccation of the lungs. At the end of the experiment, the dissected lungs and hearts were rinsed in PBS for 10–15 minutes at room temperature. They were then dehydrated in ethanol solutions (50%, 70%, 80%, 95%, and 100%; 2 hours each). After dehydration, the lungs were put into a methyl salicylate (Sigma-Aldrich) solution. When the lungs became translucent and the Microfil was visible, they were photographed using a Nikon SMZ800N stereomicroscope. The number of branches and junctions in the distal pulmonary vascular networks of all lobes was counted by ImageJ.

### Assessment of vascular remodeling.

To assess PA and PV muscularization, rat lung tissue sections (10 μm in thickness) were subjected to conventional H&E staining. The external and internal diameter of a minimum of 50 transversally cut vessels in tissue block ranging from 25 to 80 μm were measured by determining the distance between the lamina elastica externa and lumen in 2 perpendicular directions. The vessels were subdivided based on their diameter (microvessels, <50 μm and medium-sized vessels, 50–80 μm), and the assessment of muscularization was performed using ImageJ in a blinded fashion by a single researcher to reduce operator variability, which was not aware of the group allocation of the samples being analyzed. The absolute value of the medial thickness was converted to the relative value by setting the medial thickness of vehicle-treated WT rats as 1. We also assessed the muscularization of PAs and PVs by the degree of αSMA IF staining. The IF signal intensity was quantitated by ImageJ, and the result is presented as a relative signal intensity by setting the value of vehicle-treated WT rats as 1. Images were acquired using a confocal microscope.

### Immunoblot analysis.

The mice tissue lysates were prepared in lysis buffer (1% Triton X-100, 150 mM NaCl, 50 mM Tris-Cl at pH 7.5, 1 mM EDTA). The supernatants were collected, and total protein concentration was measured by NanoDrop 2000c (Thermo Fisher Scientific). Protein samples were denatured in SDS-sample buffer for 5 minutes at 95°C, loaded onto Mini-Protean TGX gels (Bio-Rad) or SurePAGE, Bis-Tris gels (GenScript) in equal amounts, and subjected to electrophoresis. Nitrocellulose membrane (Genesee Scientific) was used to blot the gels, which were blocked with 5% nonfat milk or 3% BSA in 1× Tris-buffered saline with 0.1% Tween 20 (1× TBST) for 1 hour at room temperature. The membranes were incubated at 4°C overnight with a primary antibody. Chemiluminescence signals were detected using SuperSignal West Dura extended duration substrate (Thermo Fisher Scientific) and imaged using an Odyssey Dlx Imaging System (LI-COR). Antibodies used for immunoblots are found in Supplemental Table 2. The quantity of each protein was normalized to the amount of a loading control protein. Subsequently, its relative quantity was calculated by setting the amount of the protein in the control (vehicle-treated) sample as 1.

### IP assay.

Mouse plasma samples were lysed in IP buffer (1% Triton X-100, 150 mM NaCl, 50 mM Tris-Cl at pH 7.5, 1 mM EDTA) supplemented with protease inhibitors (1:100 dilution) and phosphatase inhibitor (1:100 dilution). Lysates were nutated for 30 minutes at 4°C, followed by centrifugation at 12,000*g* for 10 minutes, and supernatants were collected. One-tenth of the lysate was saved as an input sample for immunoblotting. The lysate was incubated with indicated antibodies and anti-IgG (negative control), rotating overnight at 4°C, followed by the addition of Dynabeads Protein A/G and rocking for 4 hours at 4°C. Dynabeads were precipitated and rinsed thrice with IP buffer for 5 minutes at 4°C. The eluate was boiled at 95°C for 8 minutes in a loading buffer and immunoblotted along with input samples. For the input samples, the quantity of the indicated protein was initially normalized to the amount of a loading control protein, such as β-actin. Subsequently, its relative quantity was calculated by setting the amount of the protein in the vehicle-treated sample as 1. For the IP samples, the amount of the indicated protein in the MMC-treated sample was presented with the protein amount in the vehicle-treated sample set as 1.

### Reverse transcriptase–quantitative PCR.

Total RNA was extracted from mouse lungs and subjected to cDNA preparation by the reverse transcription reaction using an iScript cDNA Synthesis Kit (17088890, Bio-Rad). Quantitative PCR (qPCR) analysis was performed in triplicate using iQ SYBR Green Supermix (1708882, Bio-Rad). The relative expression values were determined by normalization to *GAPDH* transcript levels and calculated using the ΔΔCT method. Reverse transcriptase–qPCR (RT-qPCR) primer sequences are found in Supplemental Table 3.

### ChIP assay.

The lungs of mice treated with Veh (saline) or MMC (3 mg/kg) were harvested on day 5. Lung cells were isolated and crosslinked with 1% formaldehyde for 15 minutes at room temperature, followed by quenching with 1 M glycine. After washing the cells with 1× PBS, they were lysed using a lysis buffer containing 50 mM Tris-HCl (pH 8.1), 10 mM EDTA, 1% SDS, and a protease inhibitor. Genomic DNA was sheared to an average length of 200–500 bp by sonication, and the lysates were clarified by centrifugation at 12,000*g* for 10 minutes at 4°C. The supernatant was incubated with protein A/G Dynabeads at 4°C for 1 hour, the precleared sample was diluted 1:10 with dilution buffer (20 mM Tris-Cl pH 8.1, 150 mM NaCl, 2 mM EDTA, 1% Triton X-100, and protease inhibitor) and 1:10 volume was kept as input before incubation with nonspecific IgG (control), or an anti-ATF4 antibody overnight at 4°C followed by incubation with protein A/G Dynabeads. Next, the Dynabeads were washed with a buffer I (20 mM Tris-Cl pH 8.1, 150 mM NaCl, 2 mM EDTA, 1% Triton X-100, 0.1% SDS), buffer II (20 mM Tris-Cl pH 8.1, 500 mM NaCl, 2 mM EDTA, 1% Triton X-100, 0.1% SDS), and buffer III (10 mM Tris-Cl pH 8.1, 250 mM LiCl, 1 mM EDTA, 1% NP-40, 1% deoxycholate) at 4°C. The Dynabeads were washed twice with cold TE (10 mM Tris-Cl pH 8.1, 1 mM EDTA) and incubated in 250 μL elution buffer (200 mM NaHCO_3_, 1% SDS) at room temperature for 15 minutes. The eluates were mixed with 1:25 volume 5 M NaCl and incubated at 65°C for 4 hours. A 1:50 volume of 0.5 M EDTA, 1:25 volume of Tris-Cl pH 6.5, and proteinase K (final 100 mg/mL) were added and incubated at 45°C for 1 hour. Immunoprecipitated DNA fragments were purified with a QIAquick PCR Purification Kit (Qiagen), followed by RT-qPCR analysis. The sequences of the PCR primers for the ChIP assay are found in Supplemental Table 3.

### Isolation of PVECs.

CD31^+^ PVECs were isolated from the lung tissues by the enzymatic digestion, as previously described (45). Digestions were stopped after 20 minutes, and cells were processed into a single-cell suspension on ice. Cells were sorted with anti-CD31 magnetic beads and grown on the coverslips for IF staining.

### IF staining.

The endothelial cells isolated from mouse lung vasculature were blocked and incubated with primary antibody overnight at 4°C. Alexa Fluor secondary antibodies (Invitrogen) were applied for 2 hours at room temperature. IF images were acquired using a Leica SPE confocal or Nikon Ts2 and analyzed using ImageJ. Antibodies used are found in Supplemental Table 2.

### In vivo puromycin incorporation assay.

Mice were injected once with either a vehicle (saline) or MMC (3 mg/kg). Five days after the injection, the mice were perfused with 100 μg/mL puromycin in 1× PBS via intracardiac injection. Ten minutes following the perfusion, the lungs were harvested, and total lung lysates were subjected to SDS-PAGE. Immunoblot was conducted using an anti-puromycin antibody (Kerafast, EQ0001).

### MS sample preparation.

The mouse lung samples (50 mg) were lysed with an 8 M urea buffer supplemented with a 1× protease inhibitor cocktail (Thermo Fisher Scientific, 1861280). Cells were then homogenized via sonication, followed by centrifugation at 17,000*g* for 10 minutes at 4°C to clear the debris and extract the protein-containing supernatant. Protein concentration was measured using a BCA kit per the manufacturer’s instructions (Thermo Fisher Scientific, 23227). Proteins (100 μg each) were taken for further processing and resuspended in a digestion buffer (PreOmics, P.O.00027), followed by the addition of trypsin for in-solution peptide digestion. The digestion process was performed for 90 minutes at 37°C while shaking at 700 rpm. The resulting peptide mixture was then desalted, eluted, and dried completely using a vacuum concentrator (Labconco, 7810010). Dried peptides were reconstituted in 2% acetonitrile and 0.1% formic acid, and their concentration was measured on a NanoDrop using absorbance at 205 nm and 280 nm (Thermo Fisher Scientific). Finally, the peptide concentration was adjusted to 0.1 μg/μL for MS analysis.

### Liquid chromatography and MS analysis.

MS analysis was performed using a timsTOF Pro2 instrument (Bruker Daltonics) coupled to a nanoElute UHPLC system. Peptides were separated on a 150 μm × 25 cm C18 column (1.5 μm particle size, Bruker) at 50°C, with mobile phases comprising 0.1% formic acid in water (solvent A) and 0.1% formic acid in acetonitrile (solvent B). A 60-minute gradient was employed at a flow rate of 300 nL/min: 2% solvent B for 5 minutes, followed by a linear increase to 30% solvent B over 60 minutes, followed by column equilibration and washing. The timsTOF Pro2 was operated in parallel accumulation–serial fragmentation (DIA-PASEF) mode using data-independent acquisition settings. The MS scan range was considered from 100–1700 *m*/*z* in positive polarity with isolation widths of 25 Da. The TIMS setting was kept in custom ion mobility mode with 1/K_0_ starting at 0.7 V•s/cm^2^ to end at 1.3 V•s/cm^2^, with a frame cycle of 1.2 seconds, incorporating 120-ms ion accumulation per frame. On average, 12 PASEF scans were acquired per duty cycle. The raw LFQ spectral spectra were analyzed using DIA-NN (PMID: 31768060), a neural network-based software, to identify and quantify surface proteins. The data were searched against the UniProt-reviewed mouse proteome database. Trypsin was specified as the protease, allowing for up to one missed cleavage. Methionine oxidation and N-terminal acetylation were set as variable modifications, while cysteine carbamidomethylation was fixed. The analysis was conducted in library-free mode, with DIA-NN generating in silico spectral libraries directly from the protein sequence database. Default settings for precursor charge states and ion types were used for protein quantification. The LFQ intensities were median normalized and subsequently log-transformed. The data were normalized to total protein. Differential expression of proteins was assessed using Welch’s *t* test, with significance defined by a *P* value of less than 0.05 and a fold change (FC) threshold of greater than 1.74 or less than –1.74 to identify upregulated or downregulated proteins (46).

### MS data analysis.

Raw data files were processed with Byonic software (Protein Metrics). Fixed modifications included +113.084 C. Variable modifications included Acetyl +42.010565 N-term, pyro-Glu −17.026549 N-term Q, pyro-Glu −18.010565 N-term E. Precursor tolerance 30.0 ppm.

### MS data compilation.

Raw files were read for UniProtIDs, gene names, and their respective mappings. ‘nan,’‘’ (empty strings), and ‘2 SV’ were ignored. Mappings between gene names and UniProtIDs were not one-to-one. Some genes and UniProtIDs were unmapped. Therefore, a comprehensive, one-to-one mapping was first made before compiling the raw data. Using the Retrieve/ID mapping program at https://www.uniprot.org/ (release 2019–11), all gene names were mapped to all possible UniProtIDs, and UniprotIDs were mapped to all possible gene names. The mappings from raw files and UniProt were combined to group equivalent gene names and UniProtIDs (usually with isoforms) together. From each group, one gene name and one UniProtID were selected for downstream data compilation. Any UniProtID or gene name that was not mapped was either given a protein ID (UNM no.) or a gene name (Unm no.).

### MS data availability.

Raw proteomic data generated here are deposited in the ProteomeXchange/PRIDE repository (project accession number: PXD066782).

### Statistics.

All numerical data are presented as mean ± standard error of the mean (SEM). Statistical analysis was performed using Microsoft Excel and GraphPad Prism 10. Datasets with 2 groups were subjected to a 2-tailed Student’s *t* test, unpaired, equal variance, whereas comparison among 3 or more groups was made by ANOVA followed by Tukey’s post hoc corrections. Analysis of variance was applied to experiments with multiple parameters, 1- or 2-way, as appropriate. Significance was analyzed using a post hoc Tukey test and indicated as *P* values where required. A *P* value of less than 0.05 was considered significant.

### Study approval.

All animal experiments were conducted following the guidelines of the Institutional Animal Care and Use Committee (IACUC) of UCSF. The protocol number for the relevant animals and procedures approved by the IACUC is AN200674-00C: “Role of Growth Factor Signaling in Vascular Physiology” (approval date: April 2, 2025).

## Author contributions

AP and AH designed research studies. AP, MW, RK, AG, JL, AA, SG, PG, and AB conducted experiments and acquired data AP, MW, RK, AG, JL, AA, SG, PG, AB, APW, GL, and AH analyzed data. AP, RK, APW, BBG, GL, and AH wrote the manuscript.

## Supplementary Material

Supplemental data

Unedited blot and gel images

Supporting data values

## Version 1 08/21/2025

In-Press Preview

## Version 2 10/08/2025

Electronic publication
