# Supplementary material for: Essential role of protein kinase R in the pathogenesis of pulmonary veno-occlusive disease
Source: JCI Insight. 2025 Aug 21;10(19):e193495. doi: 10.1172/jci.insight.193495 (PMC12513476; doi:10.1172/jci.insight.193495)
Supplement: Supplemental data [file jciinsight-10-193495-s053.pdf]

Supplemental Fig. 1 Prabhakar et al.

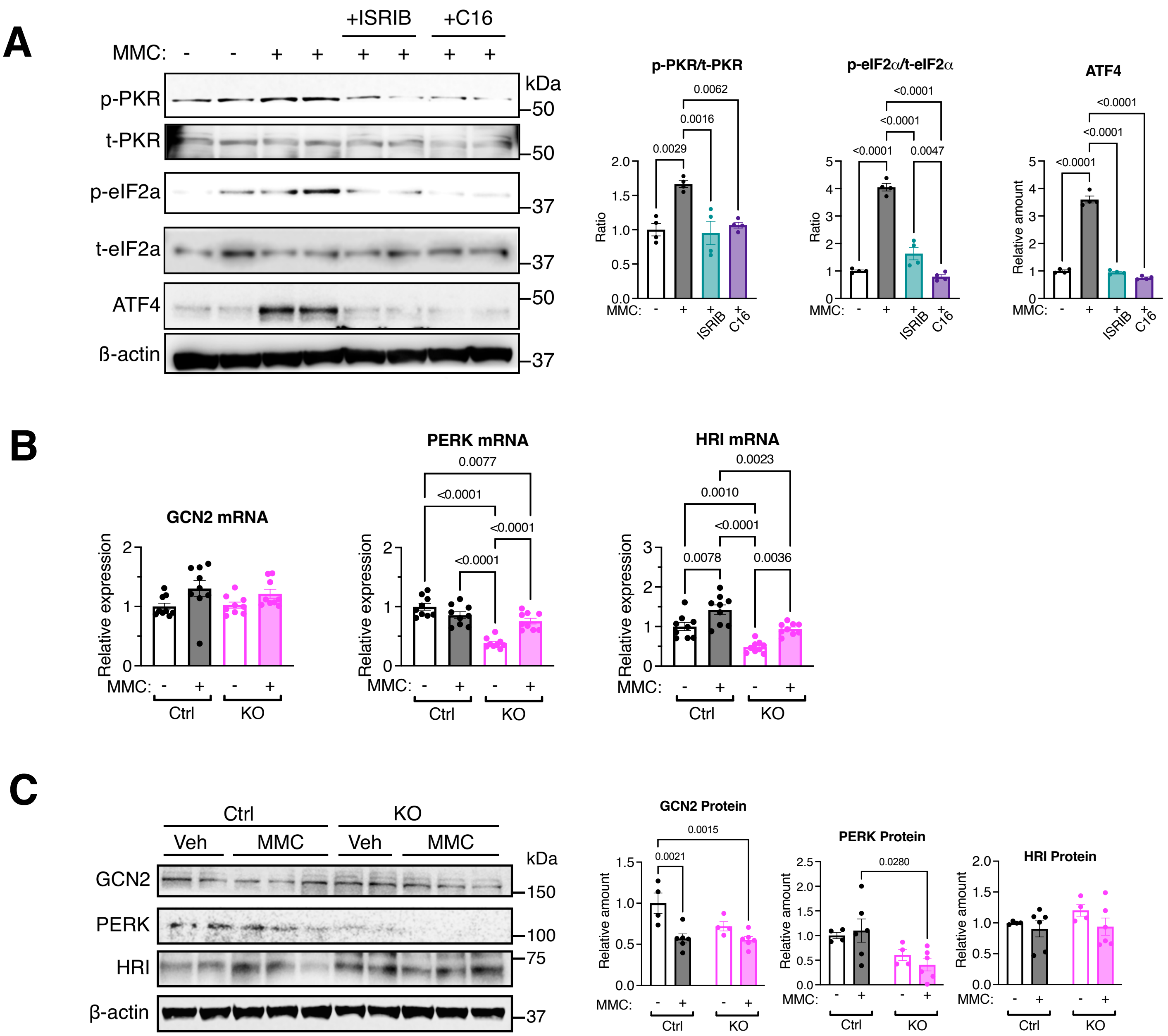

Supple. Fig. 1 MMC activates PKR but not the other three eIF2 kinases. A. Immunoblot analysis of the indicated proteins in total lung lysates from Control (Ctrl) mice co-treated with MMC and C16 (left). The relative amounts of the indicated proteins, normalized to β-actin, are shown as mean ± SEM (right). *n* = 4 samples per group. **B.** The level of mRNAs of GCN2, PERK, and HRI in the lung of Ctrl and KO mice administered with vehicle or MMC was analyzed by qRT-PCR on day 5 and shown as mean±SEM. n=6 independent samples. **C.** The amount of GCN2, PERK, HRI, and β-actin is analyzed by immunoblots (left). The relative amounts of the indicated proteins, normalized to β-actin, are shown as mean ± SEM (right). *n* = 4-6 independent samples per group. Statistical analysis was performed using one-way ANOVA with Tukey's multiple comparisons test or two-way ANOVA with Tukey's multiple comparisons test with p<0.05.

Supplemental Fig. 2 Prabhakar et al.

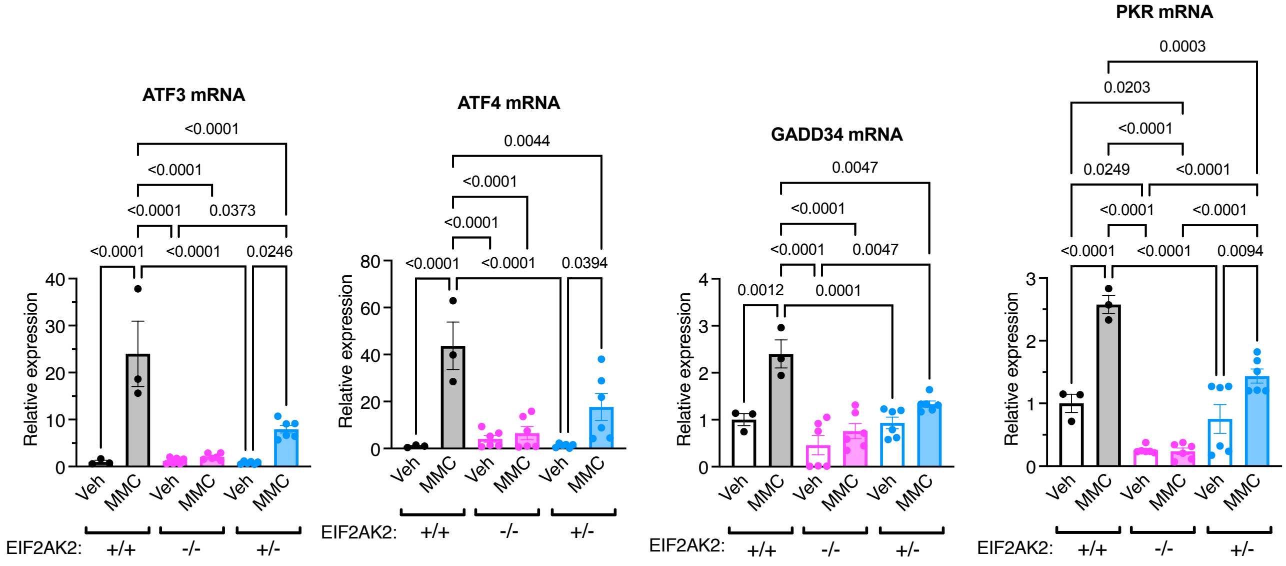

Supple. Fig. 2 The levels of ATF4 target gene transcripts are modestly increased by MMC treatment in mice heterozygous for the PKR (EIF2AK2) gene The level of mRNAs of ATF4 target genes, such as ATF3, ATF4, GADD34, and PKR in the lung of *EIF2AK2* wild type (+/+) mice (black), *EIF2AK2* homozygous-null (-/-) mice (red), and *EIF2AK2* heterozygous-null (+/-) mice (blue) administered with vehicle or MMC was analyzed by qRT-PCR on day 5 and shown as mean±SEM. n= 3-6 independent samples. Statistical analysis was performed using one-way ANOVA with Tukey's multiple comparisons test with $p<0.05$.

Supplemental Fig. 3 Prabhakar et al.

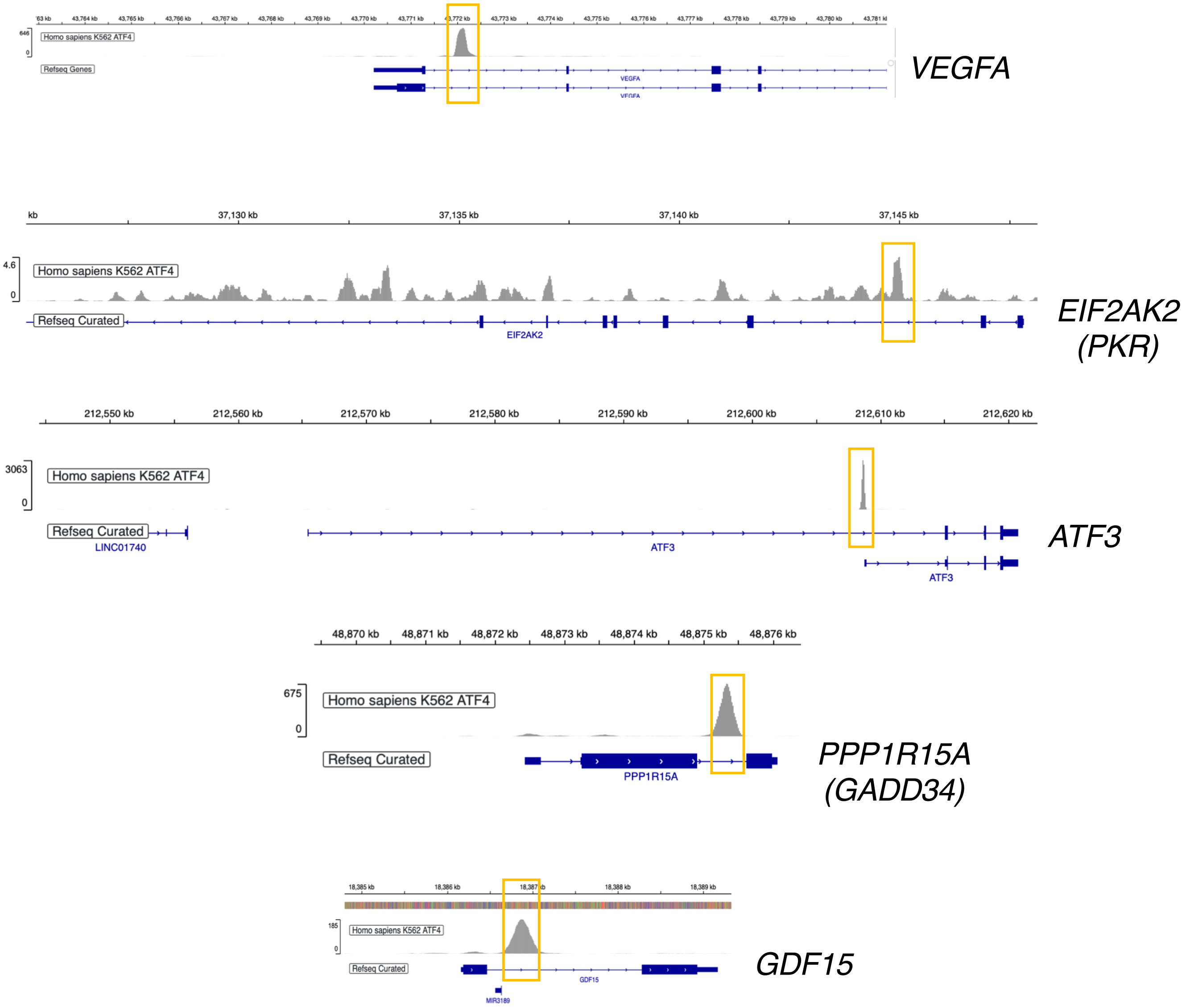

Supple. Fig. 3 ChIP-seq maps the binding sites of ATF4 in the human genome ChIP-seq data of the ENCODE database (Accession No. ENCFF484GNY and ENCFF742FPU) in human K562 cells identifies ATF4 binding sites in ATF4 target genes, such as *VEGFA*, *EIF2AK2*, *ATF3*, *PPP1R15A*, and *GDF15*. Orange rectangles indicate the genomic regions enriched by the ATF4 ChIP-seq.

Supplemental Fig. 4 Prabhakar et al.

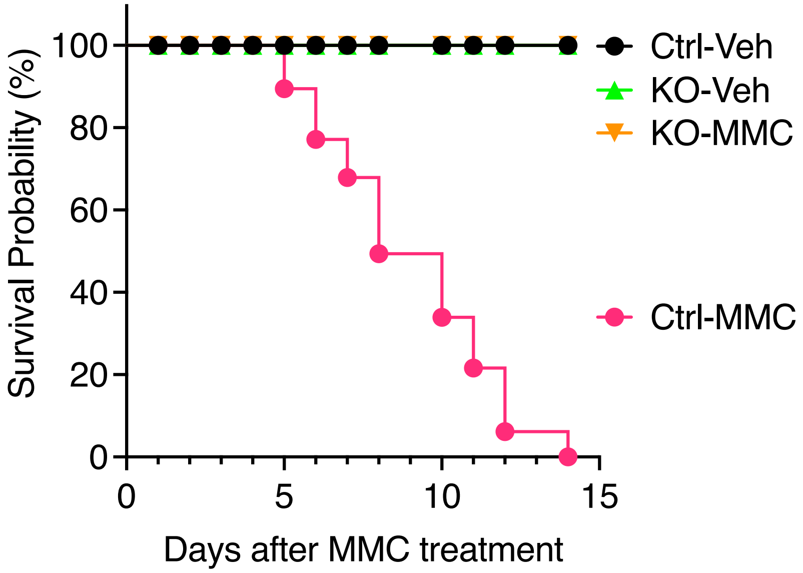

Supple. Fig. 4 Kaplan Meier Survival curve of Ctrl and KO mice following MMC treatment The survival curves of Veh-treated Ctrl (black circle), MMC-treated Ctrl (red circle), Veh-treated KO (green triangle), and MMC-treated KO mice (orange inverted triangle) are shown. These cohorts include male and female mice of 9-10 weeks old. No mortality was observed among Veh-treated Ctrl , Veh-treated KO, and MMC-treated KO mice. Statistical analysis was performed using Log-rank (Mantel-Cox) test with $p<0.05$.

Supplemental Fig. 5 Prabhakar et al.

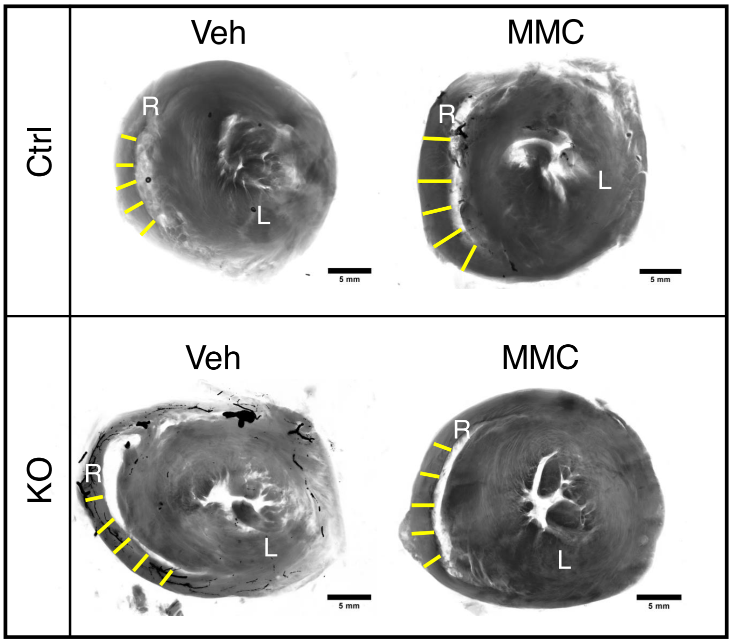

Supple. Fig. 5 KO mice do not develop right ventricular hypertrophy following MMC treatment. Representative transverse sections of hearts isolated from Veh- or MMC-treated Ctrl and KO mice are shown. The right ventricle (R) and left ventricle (V) are indicated. Wall thickness was measured as depicted by the yellow lines, and the mean values were calculated to determine the right ventricular (RV) wall thickness, as presented in Fig. 2B. Scale bar=5 mm.

Supplemental Fig. 6 Prabhakar et al.

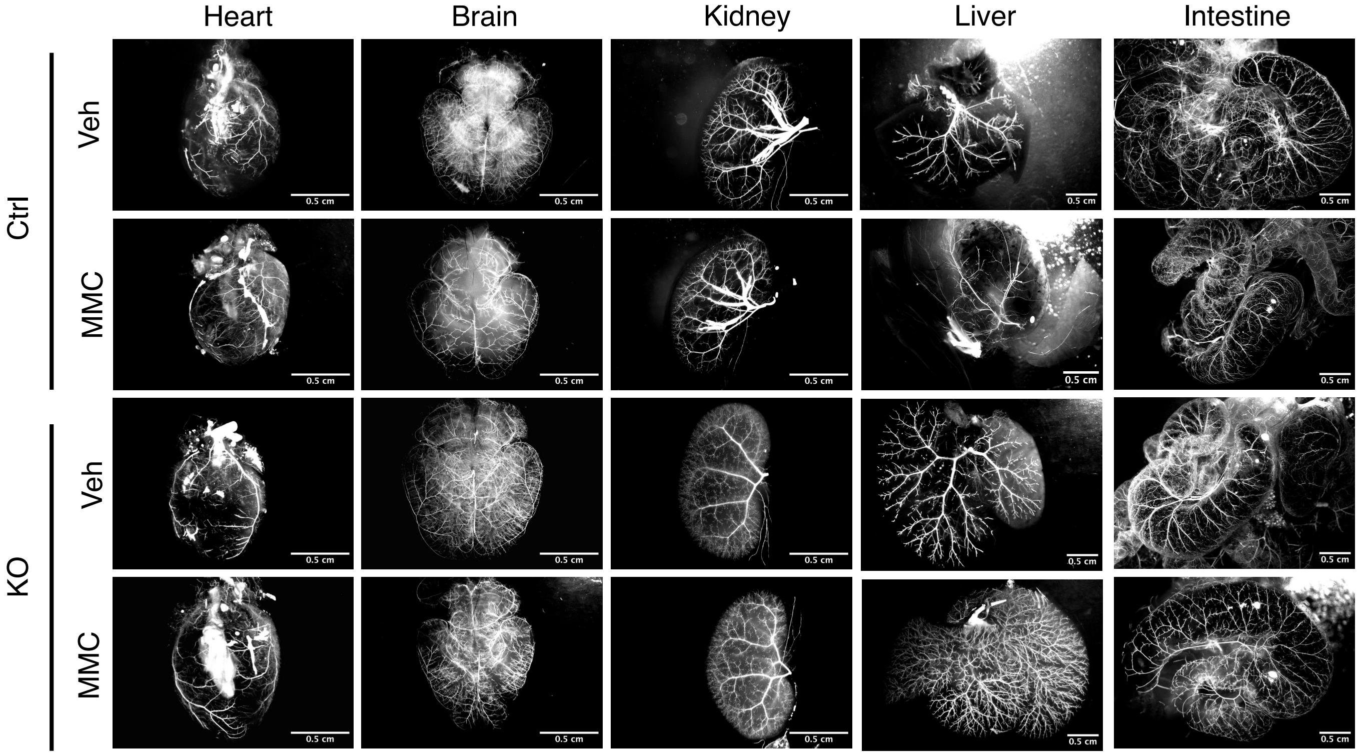

Supple. Fig. 6 MMC-induced vascular remodeling is restricted within lung Microfil casting of the vasculature in the heart, brain, kidney, liver, and intestine of Ctrl and KO mice treated with either Veh or MMC on day 5. Holistic images of the entire lung are displayed on the left, with a scale bar representing 0.5 cm. The number of branches and junctions per cm² of distal pulmonary vessels was quantified, with the data presented as mean ± SEM (right). n = 3 independent samples per group.

Supplemental Fig. 7 Prabhakar et al.

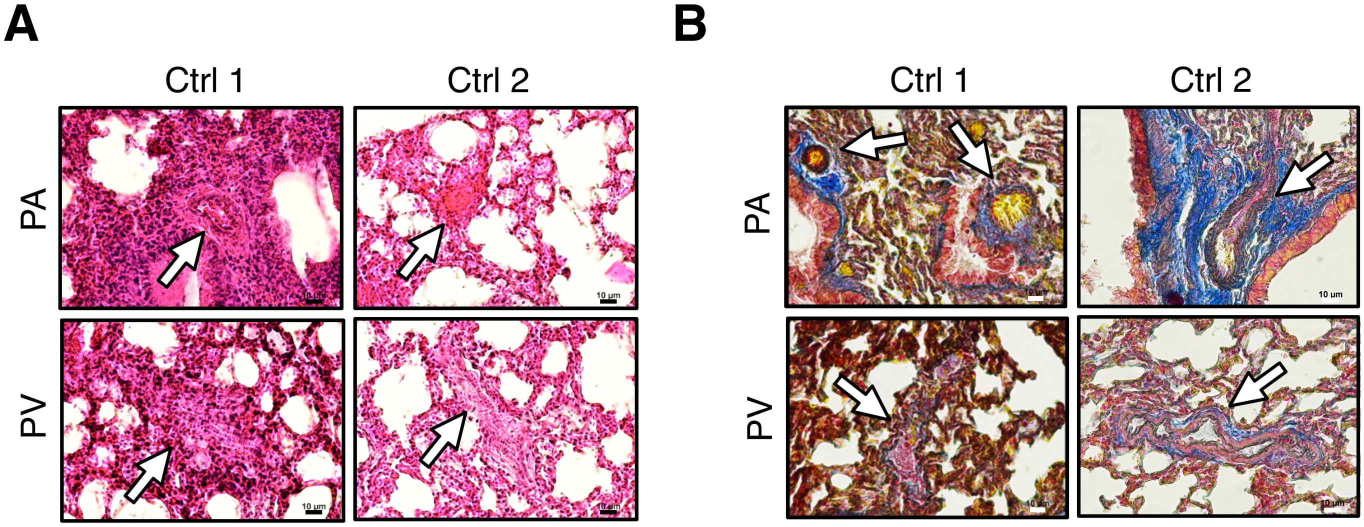

Supple. Fig. 7 Fully occluded pulmonary vessels found in MMC-treated Ctrl mice that died after MMC administration A. H&E staining images of PA and PV in two Ctrl mice (Ctrl 1 and 2) that died on day 5 following administration of MMC are shown. White arrows indicate vessels. Scale bar=10 μm. **B.** MSB staining images of PA and PV in Ctrl 1 and Ctrl 2 mice are shown. White arrows indicate vessels. White arrows indicate vessels. Collagen, smooth muscle, erythrocytes, fibrin, and platelets were visualized in blue, pink, yellow, red, and green, respectively. Scale bar=10 μm.

Supplemental Fig. 8 Prabhakar et al.

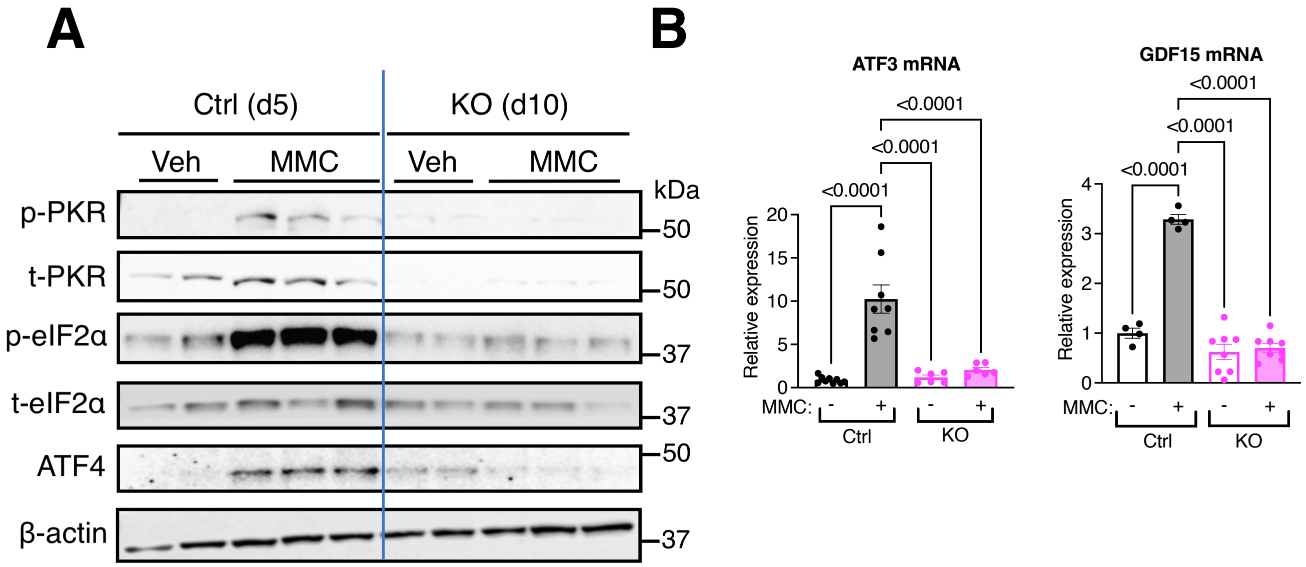

Supple. Fig. 8 No ISR activation on day 10 after the MMC treatment in PKR-null mice A. Immunoblot analysis of the indicated proteins in total lung lysates from vehicle (Veh)- or MMC-treated control (Ctrl) and PKR knockout (KO) mice. Lung tissues were collected on day 10 (d10) and representative images of the immunoblots are shown from Veh-treated Ctrl and KO mice (n = 2) and MMC-treated Ctrl and KO mice (n = 3). **B.** The levels of ATF3 and GDF15 mRNAs, target genes of ATF4, were analyzed by qRT-PCR in the lungs of Ctrl and KO mice treated with either vehicle or MMC. Samples were collected on day 5 for Ctrl mice and day 10 for KO mice and are presented as mean ± SEM. n = 4–7 independent samples per group. Statistical analysis was performed using one-way ANOVA with Tukey's multiple comparisons test with $p<0.05$.

Supplemental Fig. 9 Prabhakar et al.

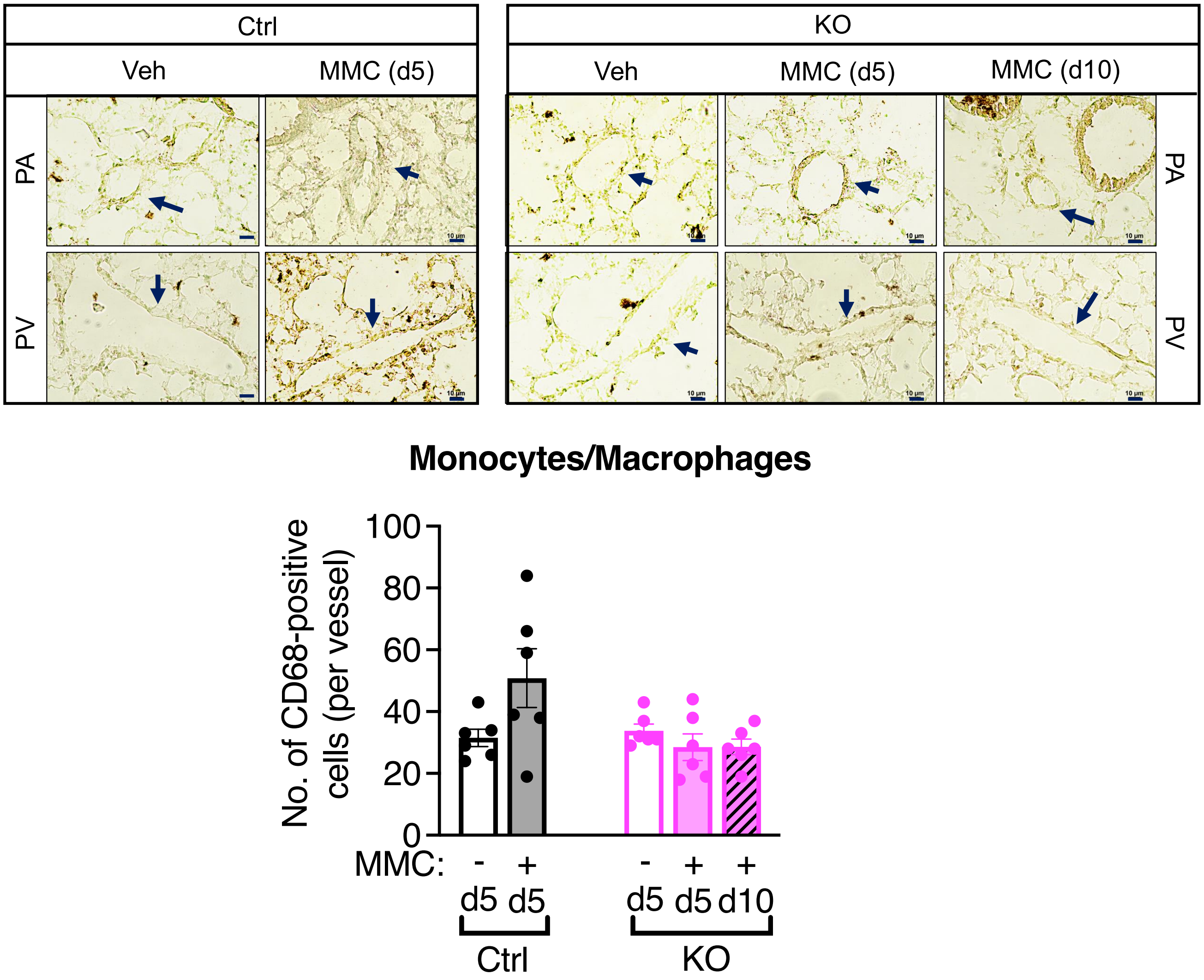

Supple. Fig. 9 Infiltration of monocytes and macrophages is observed in MMC-treated Ctrl mouse lungs, but not in KO mouse lungs CD68 immunostaining of the lungs from Ctrl on day 5 (d5) and KO mice on d5 and d10 after the administration of vehicle or MMC (top). An arrow indicates the location of the vessel (top). Scale bar=10 μm. The number of infiltrating monocytes/macrophages in the PA or PV is presented as the mean ± SEM (bottom). n=6 independent vessels. Statistical analysis was performed using two-way ANOVA with Tukey's multiple comparisons test with $p<0.05$.

Supplemental Table 1. Reagents and kits

Reagent	Company	Catalog no.
DNase I	Ambion	AM2238
Dynabeads protein A	Invitrogen	10002D
Dynabeads protein G	Invitrogen	10004D
Endothelial cell medium	ScienceCell	1001
Evans blue	Spectrum Chemicals	EV1005
Fetal Bovine Serum (FBS)	Fisher scientific	CC-4102B
Mitomycin C	Sigma-Aldrich	M0503
Mini-Protean TGX™ gels	Bio-Rad labs	4561021
SurePAGE, Bis-Tris gels	GenScript	M00665
Nitrocellulose blotting membrane	Genesee Scientific	84-875
Protease Inhibitor	Sigma	P8340
Phosphatase Inhibitor	Sigma	P5726
Preomics iST 96x	Preomics	P.O.00027
RNase inhibitors	Invitrogen	AM2696
SuperSignal™ West Dura extended duration substrate	ThermoFisher	34076
SDS-PAGE sample buffer	Invitrogen	NP0007
SDS-PAGE reducing agent	Invitrogen	NP0009
Trypsin	Life technologies	25200-072
iScript cDNA synthesis kit	Bio-Rad labs	1708891
iQ SYBR Green supermix	Bio-Rad labs	1708885

Supplemental Table 2. Antibodies

Antigen	Company	Catalog no.
ATF4	Cell signaling Technology	11815
ATF4	Santa Cruz Biotechnology	sc-390063
Alexa Flour 488 anti-rabbit IgG (H+L)	Life technologies	A21206
Alexa Flour 488 anti-mouse IgG (H+L)	Life technologies	A21202
Alexa Flour 555 anti-mouse IgG (H+L)	Life technologies	A32727
Alexa Flour 555 anti-goat IgG (H+L)	Life technologies	A21432
Alexa Flour 647 anti-mouse IgG (H+L)	Life technologies	A331571
β-actin	Sigma-Aldrich	A5441
CD68	Abcam	Ab125212
eIF2α (total)	Cell signaling Technology	9722
eIF2α (total)	Santa Cruz Biotechnology	sc-133132
Phospho-Ser51-eIF2α	Cell signaling Technology	3597
GCN2	Cell signaling Technology	3302
GADD34	Santa Cruz Biotechnology	sc-373815
HRI	Santa Cruz Biotechnology	sc-365239

PERK	Cell signaling Technology	3192
PKR (total)	Proteintech	18244-1-AP
PKR (total)	Santa Cruz Biotechnology	sc-100378
Phospho- PKR	Invitrogen	44-668G
Rad51 (D4B10)	Cell signaling Technology	8875
Rad51	Abcam	Ab133534
VE-Cadherin	Cell signaling Technology	2500
VE-Cadherin	Santa Cruz Biotechnology	sc-9989
Transferrin	Proteintech	17435-1-AP
IRDye-680RD goat anti-rabbit IgG (H+L)	Li-Cor	926-68071
IRDye-800CW goat anti-rabbit IgG (H+L)	Li-Cor	926-32211
IRDye-680RD goat anti-mouse IgG (H+L)	Li-Cor	926-68070
IRDye-800CW goat anti-mouse IgG (H+L)	Li-Cor	926-32210
anti-Rabbit-IgG-HRP-conjugated	Cell signaling Technology	7074
anti-Mouse-IgG-HRP- conjugated	Cell signaling Technology	7076

Supplemental Table 3. PCR Primers for RT-qPCR and ChIP assay

**Primer Name**	**Primer Sequence**	**Annotation**
mAtf3-qPCR-F	5’-ATAAACACCTCTGCCATCGG-3’	qRT-PCR for mouse ATF3
mAtf3-qPCR-R	5’-GCCTCCTTTTCCTCTCATCTT-3’	
mAtf4-qPCR-F	5’-ATGGCGTATTAGAGGCAGC-3’	qRT-PCR for mouse ATF4
mAtf4-qPCR-R	5’-CTTTGTCCGTTACAGCAACAC-3’	
mPkr-qPCR-F	5’-ATGCACGGAGTAGCCATTAC-3’	qRT-PCR for mouse PKR
mPkr-qPCR-R	5’-TCCTGCTTTGATCTACCTTTGG-3’	
mGadd34-qPCR-F	5’-GATCGCTTTTGGCAACCAG-3’	qRT-PCR for mouse GADD34
mGadd34-qPCR-R	5’-CAGGAGATAGAAGTTGTGGGC-3’	
mGdf15-qPCR-F	5’-GAGAGGACTCGAACTCAGAAC-3’	qRT-PCR for mouse GDF15
mGdf15-qPCR-R	5’-GACCCCAATCTCACCTCTG-3’	
mVegfa-qPCR-F	5’-GGCAGCTTGAGTTAAACGAAC-3’	qRT-PCR for mouse VEGFA
mVegfa-qPCR-R	5’-TGGTGACATGGTTAATCGGTC-3’	

mGcn2-qPCR-F	5'-ATTCGTACAGCCAAGATCCAG-3'	qRT-PCR for mouse GCN2
mGcn2-qPCR-R	5'-GTGATCCATGAACAAAGCCG-3'	
mPerk-qPCR-F	5'-TTTGAGCCAATTCAGTGCATG-3'	qRT-PCR for mouse PERK
mPerk-qPCR-R	5'-CTTCCCGCATTACCTTCTCC-3'	
mHri-qPCR-F	5'-TGTCTTTGCTGAACTCACCC-3'	qRT-PCR for mouse HRI
mHri-qPCR-R	5'-TTCTTGGAGCTCAATGGACG-3'	
mGapdh-qPCR-F	5'-GCTGGCACTGCACAAGAAGATGCG-3'	qRT-PCR for mouse GAPDH
mGapdh-qPCR-R	5'-GGGTCTGGGATGGAAATTGTGAGGG-3'	
mPkr-qPCR-F	5'-ATGTCCAGATTAGGCGTAGGT-3'	ChIP for mouse PKR
mPkr-qPCR-R	5'-AAATGTCCCCAGGAGAAAGGA-3'	
mAtf3-qPCR-F	5'-CTGAAGGCCGAGAGGTCTCCG-3'	ChIP for mouse ATF3
mAtf3-qPCR-R	5'-GTCAGCCAGAGCACAGCAAGT-3'	
mGadd34-qPCR-F	5'-GCGTGGACGATGTTGGCGCAG-3'	ChIP for mouse GADD34
mGadd34-qPCR-R	TAGCAAAGGCTGTCCCGGCCG-3'	
mGdf15-qPCR-F	5'-ACGGAAGAACCTGCGGGAA-3'	ChIP for mouse GDF15
mGdf15-qPCR-R	5'-CCTCCCATCCAAGCGACTGT-3'	
mVegfa-qPCR-F	5'-CTAGCTTGTTGGGCCACCTGCA-3'	ChIP for mouse VEGFA
mVegfa-qPCR-R	5'-GCTGGGGATACCTCCAGAGGTC-3'	

Supplemental Table 4. Instruments and software

**Instrument/ software**	**Experiment**	**Company**	**Model no./ version no.**
LI-COR	Immunoblot	Odyssey	Odyssey Dlx Imaging System
Dismembrator/Sonicator	Sonication	Fisher Scientific	550 sonic dismembrator
Tissue lyser	Tissue lysis	Qiagen	TissueLyser II
RT-PCR machine	qRT-PCR	BioRad	CFX connect
NanoDrop spectrometer	Protein, DNA, and RNA quantitation	Thermo Scientific	NanoDrop 2000c
Confocal Microscope	Immunofluorescence imaging	Leica	Leica SPE

Inverted Phase Contrast Microscope	Immunofluorescence imaging	Nikon	Eclipse TS2
Digital Color Microscope Camera	Attached to Eclipse TS2	Nikon	DS-Fi3
Stereoscope	Microifil casting imaging	Nikon	SMZ800N
Rat Ventilator	RV catheterization	Harvard Apparatus	VentElite
1.4F Pressure-volume catheter	Hemodynamics measurement	Millar AD Instruments	SPR-839
Hemodynamics analysis software	Hemodynamics data analysis	AD Instruments	LabChart 8
MS Excel	Statistical analysis	Microsoft	MS office 365
GraphPad Prism	Statistical analysis	GraphPad	Prism 10
Cell sorter	Cell sorting	Miltenyi Biotec	autoMACS NEO
timsTOF Pro2 and nanoElute UHPLC	Mass Spectrometry	Bruker Daltonics	Pro2
