# Supplementary material for: Essential role of protein kinase R in the pathogenesis of pulmonary veno-occlusive disease
Source: JCI Insight. 2025 Aug 21;10(19):e193495. doi: 10.1172/jci.insight.193495 (PMC12513476; doi:10.1172/jci.insight.193495)
Supplement: Unedited blot and gel images [file jciinsight-10-193495-s054.pdf]

Fig. 1A Unprocessed blots

Prabhakar et al

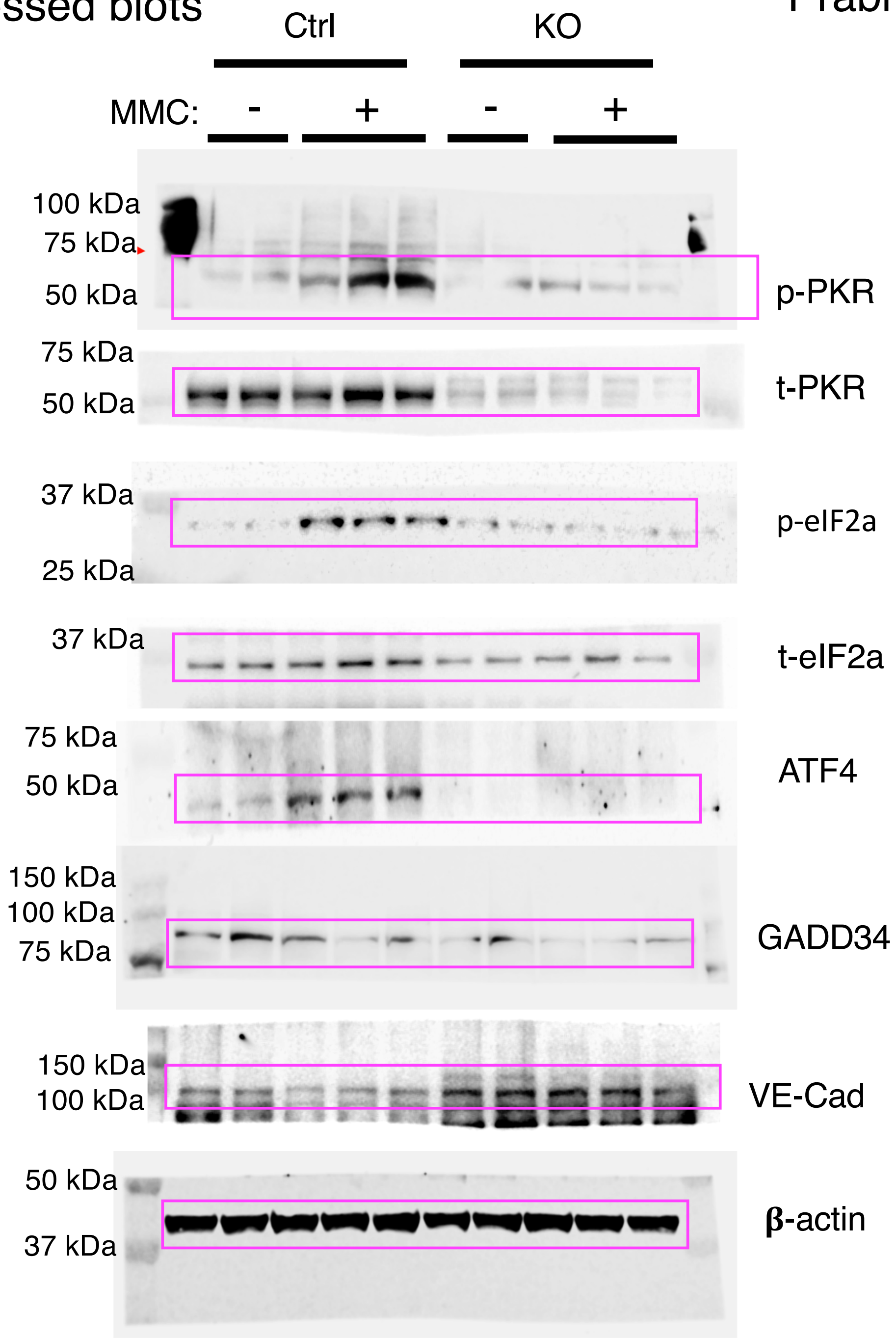

Fig. S1A Unprocessed blots

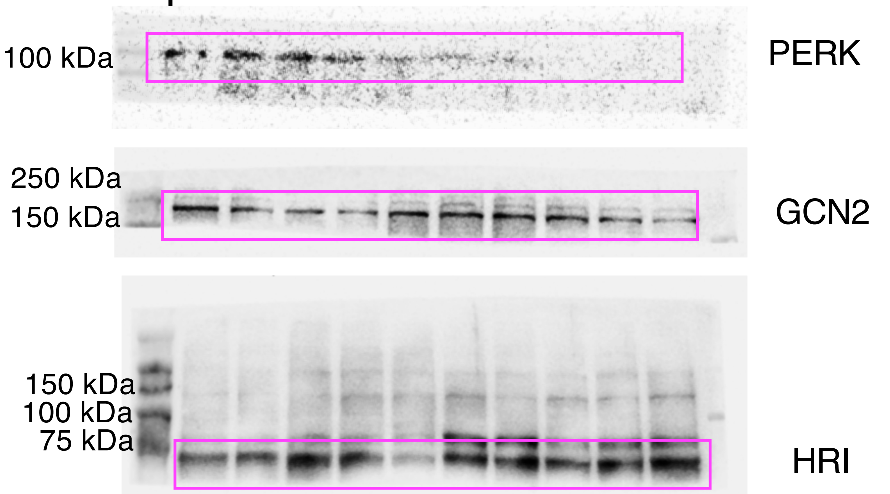

Bands from the magenta frame were used for this paper

Fig. 1D Unprocessed blots

Prabhakar et al

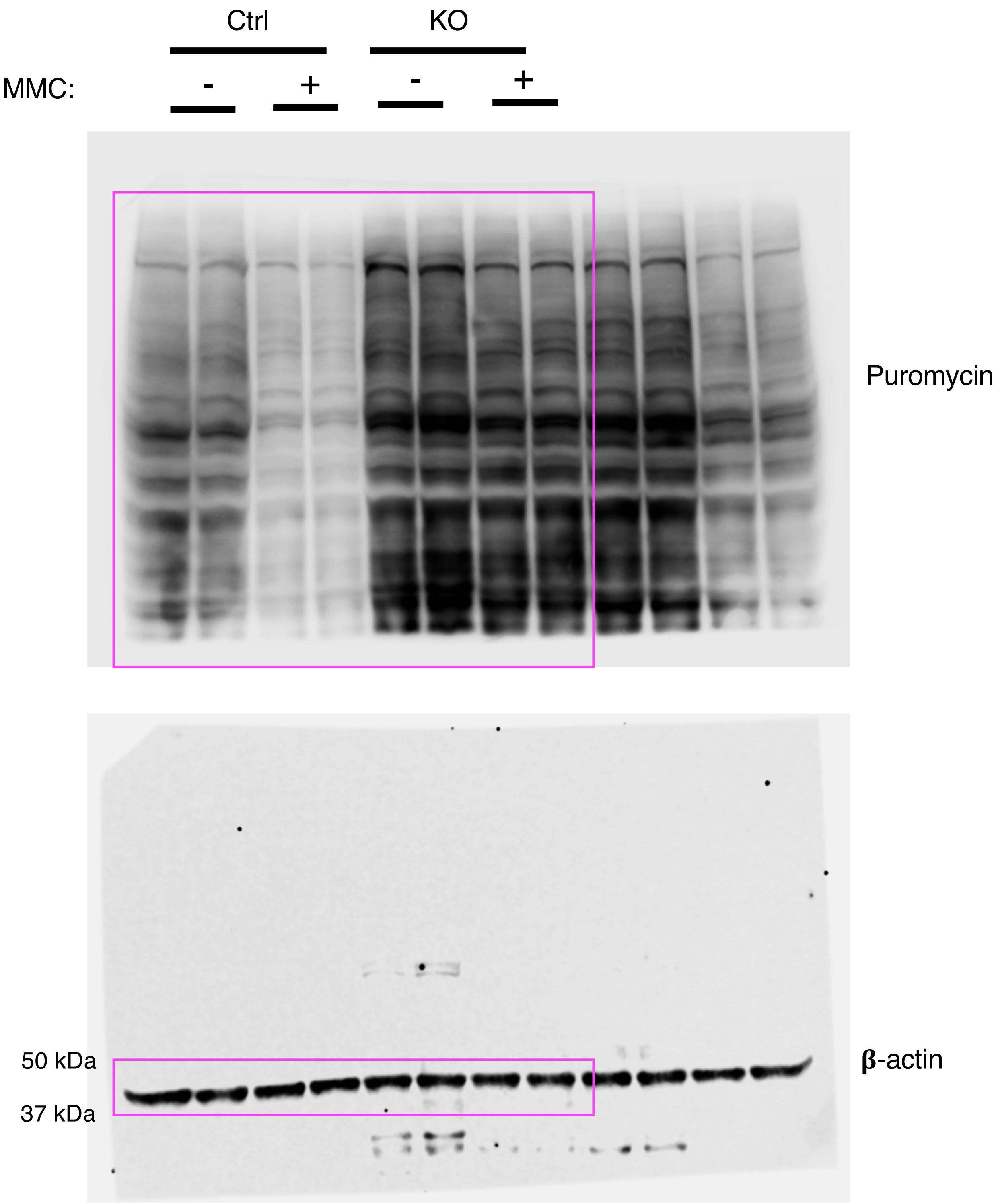

Bands from the magenta frame were used for this paper

Fig. 3C Unprocessed blots

Prabhakar et al

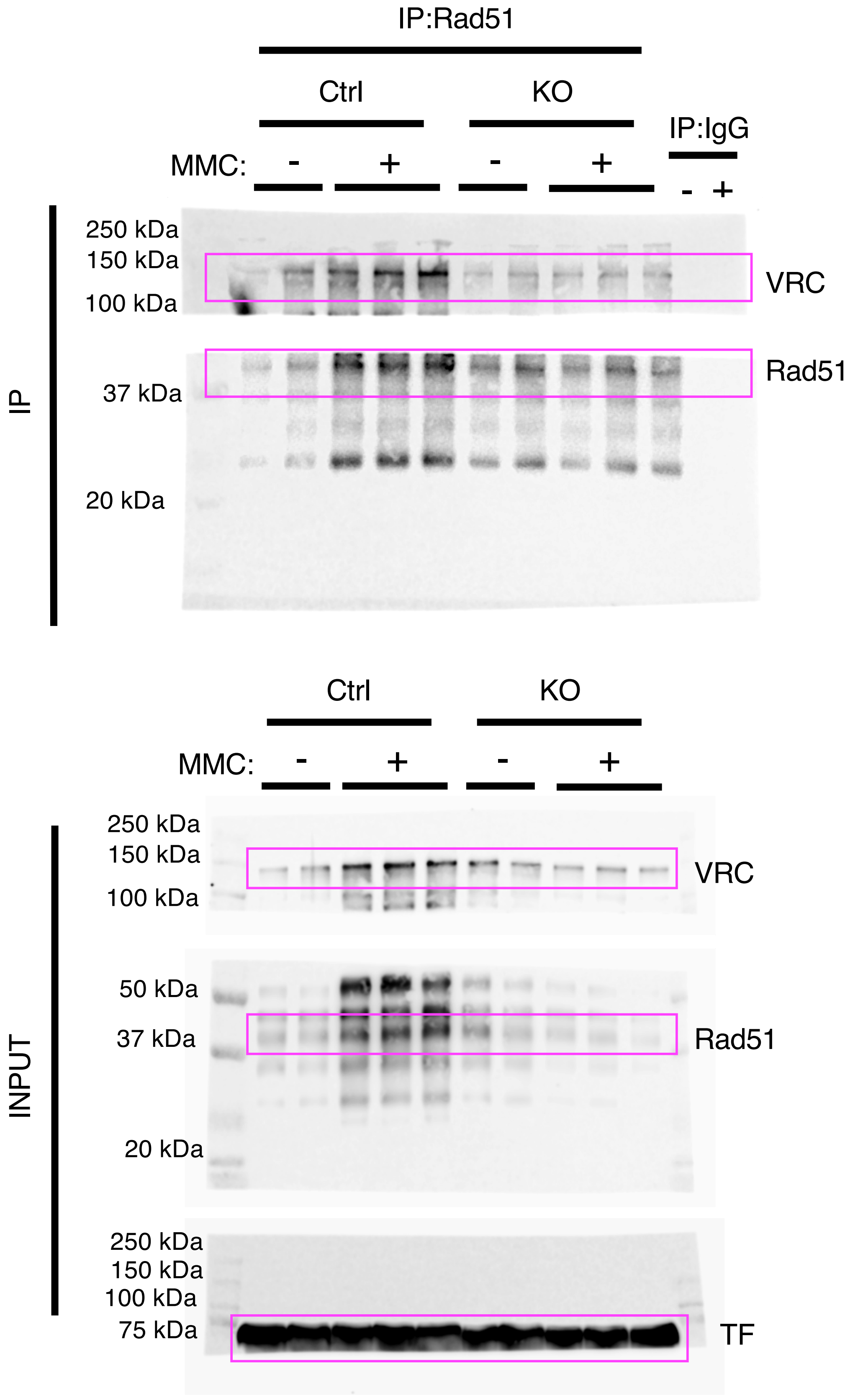

Bands from the magenta frame were used for this paper

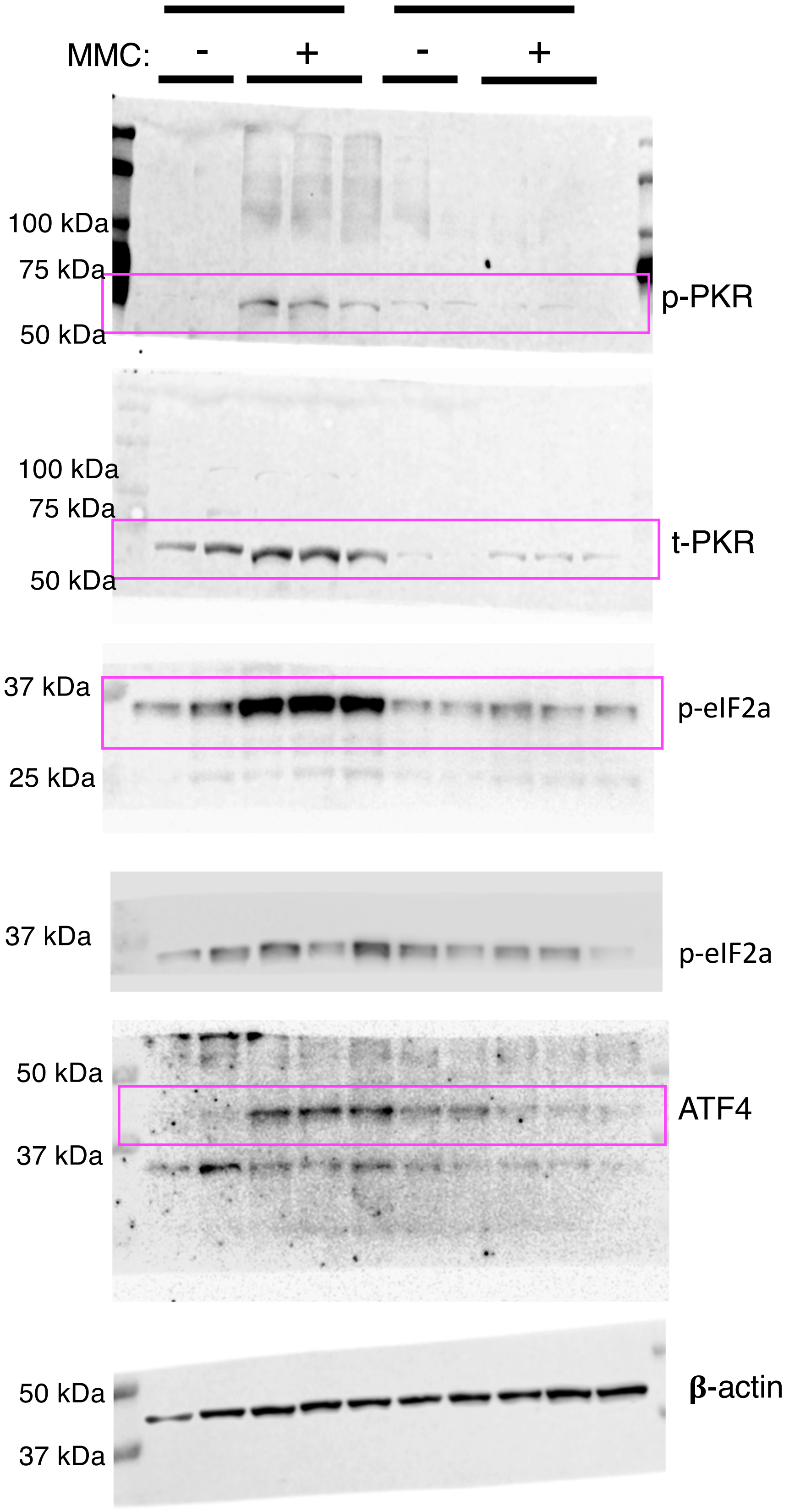

Bands from the magenta frame were used for this paper
